# Extracellular Matrix Profiling and Disease Modelling in Engineered Vascular Smooth Muscle Cell Tissues

**DOI:** 10.1016/j.mbplus.2022.100122

**Published:** 2022-09-17

**Authors:** Ella Reed, Adam Fellows, Ruifang Lu, Marieke Rienks, Lukas Schmidt, Xiaoke Yin, Elisa Duregotti, Mona Brandt, Susanne Krasemann, Kristin Hartmann, Javier Barallobre-Barreiro, Owen Addison, Friederike Cuello, Arne Hansen, Manuel Mayr

**Affiliations:** a King's British Heart Foundation Centre, School of Cardiovascular and Metabolic Medicine and Sciences, London SE5 9NU, UK; b National Heart and Lung Institute, Imperial College London, Du Cane Road, London W12 0NN, UK; c Institute of Experimental Pharmacology and Toxicology, University Medical Center Hamburg-Eppendorf, 20246 Hamburg, Germany; d German Center for Cardiovascular Research (DZHK), Partner Site Hamburg/Lübeck/Kiel, University Medical Center Hamburg-Eppendorf, Germany; e Institute of Neuropathology, University Medical Center Hamburg-Eppendorf, 20246 Hamburg, Germany; f Centre of Oral, Clinical & Translational Sciences, Faculty of Dentistry, Oral & Craniofacial Sciences, King’s College London, Guy’s Hospital, London SE1 9RT, UK

**Keywords:** Tissue engineering, 3D cell culture, Proteomics, Smooth muscle cells, ECM, Calcification, 2D, Two-dimensional, 3D, Three-dimensional, ECM, Extracellular matrix, SMC, Smooth muscle cell, EVT, Engineered vascular smooth muscle cell tissue, EHT, Engineered heart tissue, TCP, Tissue culture polystyrene, ADAMTS, A disintegrin and metalloproteinase with thrombospondin motifs, TGFβ-1, Transforming growth factor beta-1, ALKi, Activin-like kinase inhibitor, LC-MS/MS, Liquid chromatography with tandem mass spectrometry

## Abstract

Aortic smooth muscle cells (SMCs) have an intrinsic role in regulating vessel homeostasis and pathological remodelling. In two-dimensional (2D) cell culture formats, however, SMCs are not embedded in their physiological extracellular matrix (ECM) environment. To overcome the limitations of conventional 2D SMC cultures, we established a 3D *in vitro* model of engineered vascular smooth muscle cell tissues (EVTs). EVTs were casted from primary murine aortic SMCs by suspending a SMC-fibrin master mix between two flexible silicon-posts at day 0 before prolonged culture up to 14 days. Immunohistochemical analysis of EVT longitudinal sections demonstrated that SMCs were aligned, viable and secretory. Mass spectrometry-based proteomics analysis of murine EVT lysates was performed and identified 135 matrisome proteins. Proteoglycans, including the large aggregating proteoglycan versican, accumulated within EVTs by day 7 of culture. This was followed by the deposition of collagens, elastin-binding proteins and matrix regulators up to day 14 of culture. In contrast to 2D SMC controls, accumulation of versican occurred in parallel to an increase in versikine, a cleavage product mediated by proteases of the A Disintegrin and Metalloproteinase with Thrombospondin motifs (ADAMTS) family. Next, we tested the response of EVTs to stimulation with transforming growth factor beta-1 (TGFβ-1). EVTs contracted in response to TGFβ-1 stimulation with altered ECM composition. In contrast, treatment with the pharmacological activin-like kinase inhibitor (ALKi) SB 431542 suppressed ECM secretion. As a disease stimulus, we performed calcification assays. The ECM acts as a nidus for calcium phosphate deposition in the arterial wall. We compared the onset and extent of calcification in EVTs and 2D SMCs cultured under high calcium and phosphate conditions for 7 days. Calcified EVTs displayed increased tissue stiffness by up to 30 % compared to non-calcified controls. Unlike the rapid calcification of SMCs in 2D cultures, EVTs sustained expression of the calcification inhibitor matrix Gla protein and allowed for better discrimination of the calcification propensity between independent biological replicates. In summary, EVTs are an intuitive and versatile model to investigate ECM synthesis and turnover by SMCs in a 3D environment. Unlike conventional 2D cultures, EVTs provide a more relevant pathophysiological model for retention of the nascent ECM produced by SMCs.

## Highlights

• Engineered vascular smooth muscle cell tissues (EVTs) are a novel 3D culture model.
• Unlike in 2D cultures, the nascent ECM is retained within EVTs.
• This is the first proteomics description of the ECM in a 3D SMC culture model.
• EVTs may be useful for pre-clinical vascular drug screening.
• EVTs can be employed as a 3D *in vitro* calcification model.

## Graphical abstract

**Figure:**
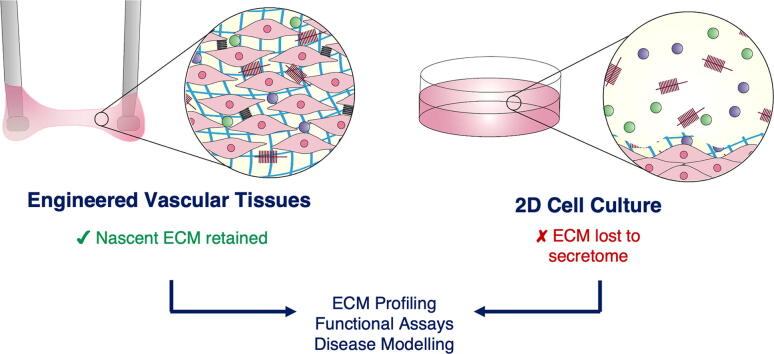

## Introduction

Atherosclerosis is the leading cause of cardiovascular disease [1]. The aetiology of atherosclerosis involves the sequential and progressive modification of the arterial wall [2]. Alongside deposition of lipoproteins [3], inflammation and calcification are key features of atherosclerotic vessels [4]. In atherosclerosis, aortic smooth muscle cells (SMCs) dedifferentiate from a quiescent and contractile state to displaying features of other cell types [5], [6]. These SMC-derived phenotypes have been identified *in vivo* utilising state-of-the-art single cell RNA sequencing and lineage tracing technologies and include proinflammatory macrophages, secretory fibroblasts, and calcifying osteoblasts [7], [8], [9], [10], [11]. Consequently, SMC phenotype switching is associated with atherosclerosis [12].

SMC phenotype switching is accompanied with altered expression and turnover of their surrounding extracellular matrix (ECM). This is crucial as the SMC-derived ECM consists of over 300-affiliated and secreted proteins that can contribute to the functionality of the arterial wall [13]. Elastin and fibrillar collagens are the most well-known of these and contribute to arterial wall compliance and resistance [14]. In atherosclerosis, secretion of ECM collagens by dedifferentiated SMCs may be protective by contributing to plaque stability [15]. However, beyond elastin and collagens, ECM proteoglycans are also important in arterial wall homeostasis and pathophysiology. The large aggregating proteoglycans aggrecan and versican contribute to vessel hydration and turgidity [16], [17], [18], while small leucine-rich repeat proteoglycans affect collagen fibril formation as well as growth factor signalling [19], [20]. Both types of proteoglycans have been previously associated with atherosclerosis [21], [22], [23], [24]. Proteoglycans, including versican, are key players in the development of atherosclerosis by contributing to vascular retention of charged lipoprotein particles [24]. In addition to altered abundance of ECM proteins, post-translational modifications and cleavage by their proteases provides added diversity to the extracellular proteome across different vascular diseases [25], [26]. For example, ECM degradation can contribute to plaque destabilisation in atherosclerosis [27].

The role of SMCs and their ECM in atherosclerosis is therefore dynamic and functionally ambivalent [5]. Currently, researchers are reliant on two-dimensional (2D) cell cultures to investigate SMC behaviours *in vitro*. However, standard 2D cell culture formats fail to recapitulate the *in vivo* niche including 3D cell-ECM interactions [28]*.* Additionally, tissue culture polystyrene (TCP) displays a non-physiological substrate stiffness of GPa compared to MPa in the vessel wall [29] and may contribute to SMC dedifferentiation [30] and altered ECM secretion [31]. Novel 3D cell culture models may alleviate at least some of the limitations associated with traditional 2D cultures. Notably, tissue engineered vascular medias [32] and engineered vascular grafts [33], alongside other innovative models, have been developed. However, there remains a need for a standardised 3D SMC culture model that is cost-effective, scalable and reproducible.

In this work, we propose engineered vascular smooth muscle cell tissues (EVTs) as a novel 3D cell culture model that meets these requirements. EVTs are casted using primary murine aortic SMCs and cultured for up to 14 days. For the first time, the extracellular proteome of a 3D SMC culture model has been interrogated by a discovery proteomics approach. We demonstrate how the retained ECM within EVTs reflects *in vivo* protein diversity, is dynamic and responsive to disease stimuli. Furthermore, EVTs reflect key aspects of SMC behaviour: EVTs remodel and contract in response to transforming growth factor beta-1 (TGFβ-1), while calcified EVTs become stiff and bone-like.

## Results

### Formation and characterisation of EVTs

**Fig. 1:**
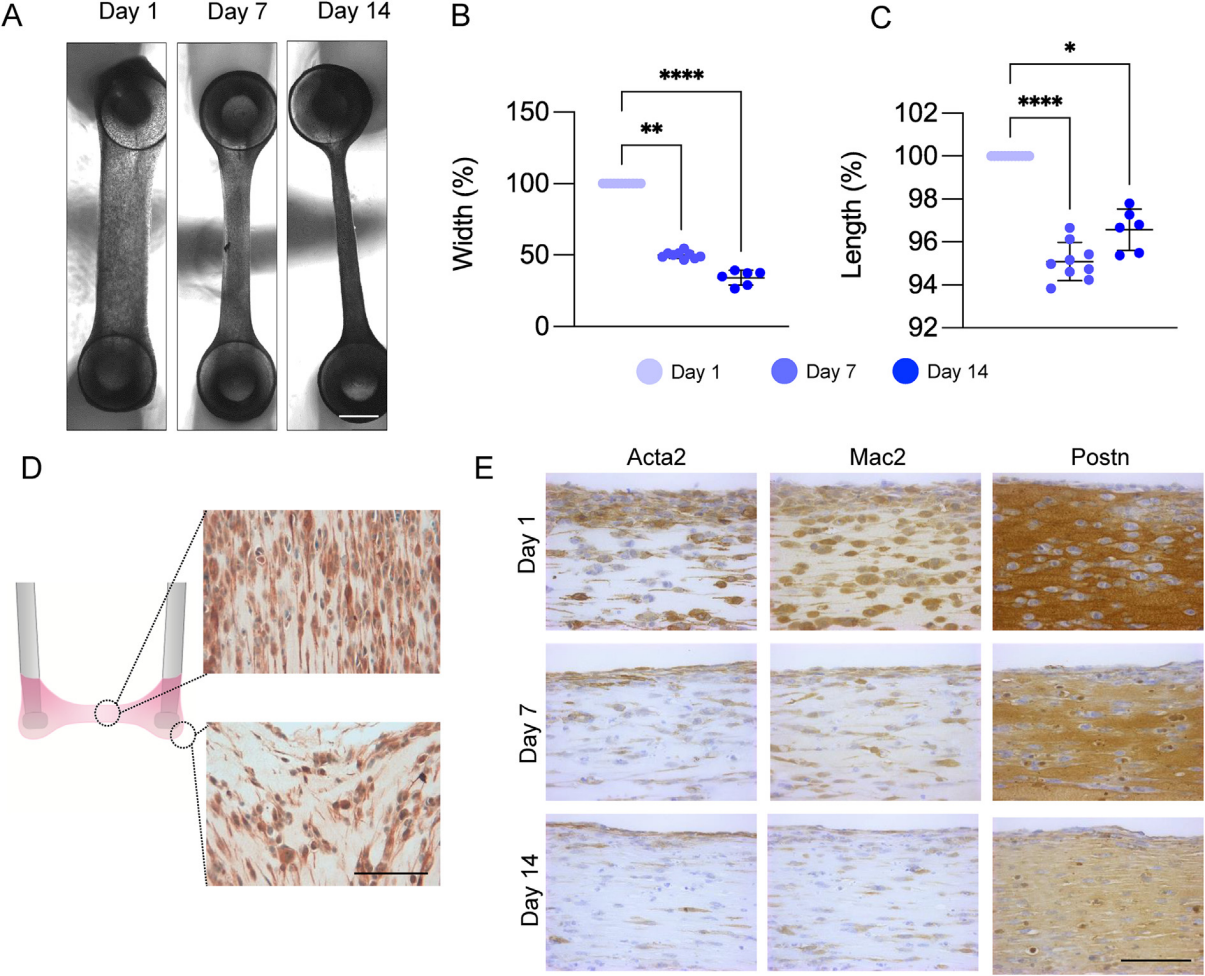
**Physical and molecular remodelling of murine EVTs.** (**A**) Inverted light microscope images of murine SMC EVTs after 1, 7 and 14 days of culture. Scale bar = 1 mm. Quantification of murine EVT width (**B**) and length (**C**) after 1, 7 and 14 days of culture. Significance was determined by one-way ANOVA with Dunn’s correction for multiple comparisons. A p value < 0.05 was considered significant. (**D**) Representative images of transverse murine EVT sections stained for transgelin (Tagln) show cell alignment at the centre of the construct and around the posts of EVTs after 7 days of culture. Scale bar = 100 μm. (**E**) Representative images of longitudinal sections of murine EVTs stained for smooth muscle actin (Acta2), galectin-3 (Mac2 / Lgals3) and periostin (Postn) after 1, 7 and 14 days of culture. Scale bar = 100 μm.

EVTs were casted using an adapted protocol for the formation of engineered heart tissues (EHTs) [34]. Addition of thrombin to a master mixture containing bovine fibrinogen and primary murine SMCs allowed for the rapid formation of fibrin and embedment of SMCs within the 3D fibrin hydrogel (Supplementary Fig.1). EVTs were cultured for 1, 7 or 14 consecutive days before harvesting. Inverted light microscopy revealed how EVTs became shorter and thinner with prolonged time in culture (Fig. 1**A-C;** Supplementary Fig.2). The spatial localisation of SMCs was investigated using immunohistochemistry of longitudinal murine EVT sections. SMCs within EVTs were initially rounded at day 1, however, after prolonged culture SMCs became elongated. This effect may be attributed to suspension of the tissue construct between two polydimethylsiloxane (PDMS) posts resulting in SMCs at the centre of the construct being aligned along the longitudinal axis (Fig. 1**D**). Conversely, at the posts SMCs appeared disordered. Compared to conventionally cultured 2D SMCs which exhibit low organisation in culture, SMCs within EVTs demonstrated a more physiological tissue-like architecture (Supplementary Fig.3). SMCs cultured within EVTs displayed reduced abundance of the contractile marker alpha smooth muscle actin (Acta2) with prolonged culture (Fig. 1**E**). Inflammation and injury associated proteins including galectin-3 (Mac2 / Lgals3) [7] and periostin (Postn) [35] gradually reduced over time (Fig. 1**E**). Immunohistochemical staining for human SMCs within EVTs are presented in Supplementary Fig.4.

### ECM remodelling in EVTs

**Fig. 2:**
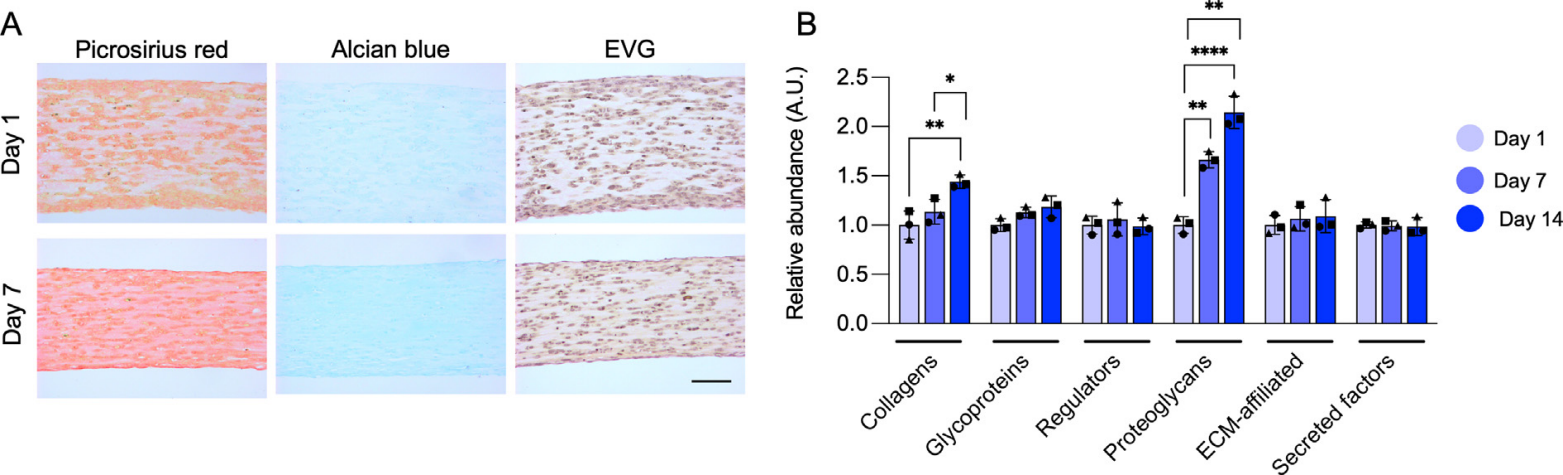
**Collagen and proteoglycan accumulation in murine EVTs.** (**A**) Representative images of transverse murine EVT sections stained for Picrosirius red (collagens), Alcian blue (glycosylated proteins), and Elastin van Gieson (EVG) stain (elastin) after 1 and 7 days of culture. Scale bar = 100 μm. (**B**) Total abundances of ECM protein groups in EVTs cultured for 1, 7 or 14 days detected by LC-MS/MS. Results are normalised to the respective EVT day 1 abundance. Shapes (circle, square, triangle) indicate independent SMC isolates. Error bars show SD. Significance was determined by one-way ANOVA with Dunn’s correction for multiple comparisons. A p value < 0.05 was considered significant.

Limitation of 2D SMC cultures is the loss of ECM to the surrounding media. In contrast, the nascent ECM is retained within the EVT environment. The collagen content of murine EVT sections was visualised using Picrosirius red staining, whereas Alcian blue staining was used to visualise the presence of proteoglycans (Fig. 2**A**). While collagens and proteoglycans were present in abundance in day 7 EVTs little staining was observed for elastin.

**Fig. 3:**
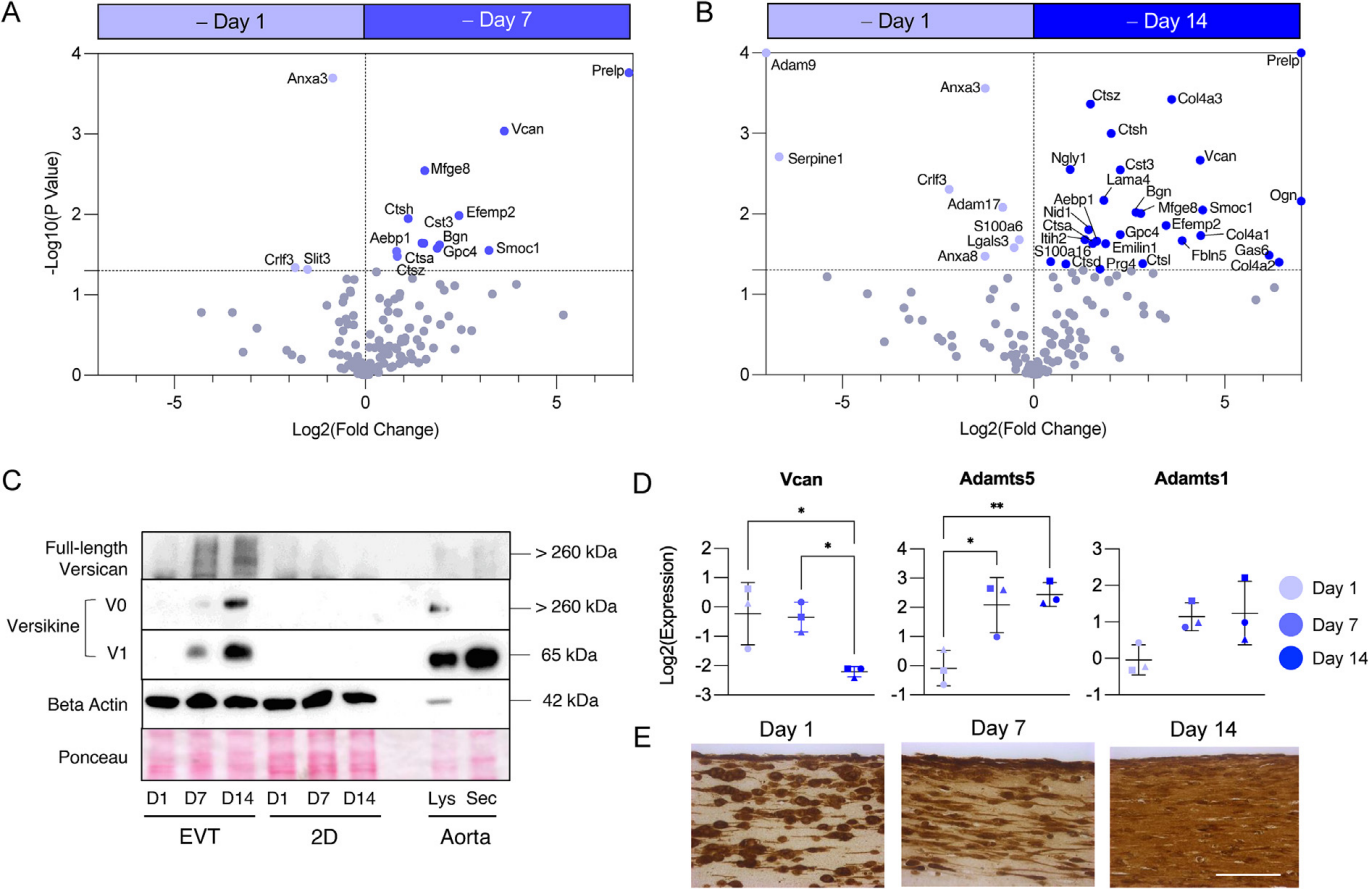
**ECM remodelling in murine EVTs.** The volcano plots indicate ECM accumulation in EVTs after 7 (**A**) or 14 (**B**) days of culture compared to day 1. N = 3 biological replicates and N = 2 technical replicates per condition. Significance was determined using an unpaired Student’s t-test with a p value < 0.05 considered significant. Proteins with a log2(fold change) > 7 or a log10(p value) > 4 were imputed as 7 and 4, respectively. (**C**) Western blotting confirmed accumulation of full-length versican (Vcan) and an ADAMTS-mediated versican cleavage product, versikine, in EVTs compared to 2D lysates after 1, 7 or 14 days of culture. Western blot lanes for EVTs and 2D SMCs cultured for 1, 7 and 14 days (pools of N = 3 biological replicates). The final two lanes are protein extracts from murine aortic lysates (Lys) and secreted (Sec) proteins by explanted murine aortas in organ cultures. Beta actin (ActB) and Ponceau staining were used as loading controls. (**D**) Versican (Vcan), Adamts5, and Adamts1 transcript expression in murine EVTs after 1, 7 and 14 days of culture. Shapes (circle, square, triangle) indicate independent SMC isolates. Error bars show SD. Significance was determined by one-way ANOVA with Dunn’s correction for multiple comparisons, a p value < 0.05 was considered significant. (**E**) Immunohistochemistry reveals versikine accumulation within EVTs cultured up to 14 days. Versikine is generated by ADAMTS-mediated cleavage of versican. Scale bar = 100 μm.

For a more detailed investigation into the ECM composition discovery proteomics analysis of murine EVT lysates at day 1, 7 and 14 of culture was performed. The proteomics findings confirmed the histology findings: ECM proteins predominantly belonging to the collagen and proteoglycan families accumulated within EVTs up until day 14 (Fig. 2**B**). The volcano plots depicted in Fig. 3**A and 3B** illustrate the ECM proteins dysregulated in EVTs at day 7 and 14 of culture compared to day 1. Compared to day 14, day 1 EVTs displayed upregulation of galectin-3 (Mac2 / Lgals3), plasminogen activator inhibitor 1 (Serpine1), and a disintegrin and metalloproteinase domain-containing protein 9 (Adam9). EVTs cultured for 7 and 14 days displayed a significant increase in ECM proteoglycans (Vcan, Bgn, Prelp, Ogn, Prg4), elastin-binding proteins (Efemp2, Lama4, Emilin1) and basement membrane proteins (Col4a1, Col4a2, Col4a3, Nid1, Gpc4).

The large aggregating proteoglycan versican (Vcan) was consistently amongst the most significantly upregulated proteins after prolonged EVT culture. Upregulation of Vcan in EVTs was confirmed using immunoblotting (Fig. 3**C**). In addition to accumulation of full-length Vcan, we demonstrated the retention of versikine [36], a cleavage product of Vcan mediated by members of the ADAMTS (A Disintegrin and Metalloproteinase with Thrombospondin motifs) protease family. While Vcan transcript expression was significantly reduced with prolonged EVT culture (Fig. 3**D**) transcript expression of Adamts5, the main ADAMTS member responsible for versican cleavage in mice [24], was significantly increased. The accumulation of versikine in EVTs over time was confirmed using immunohistochemistry (Fig. 3**E**).

### Contraction of EVTs in response to TGFβ-1 stimulation

**Fig. 4:**
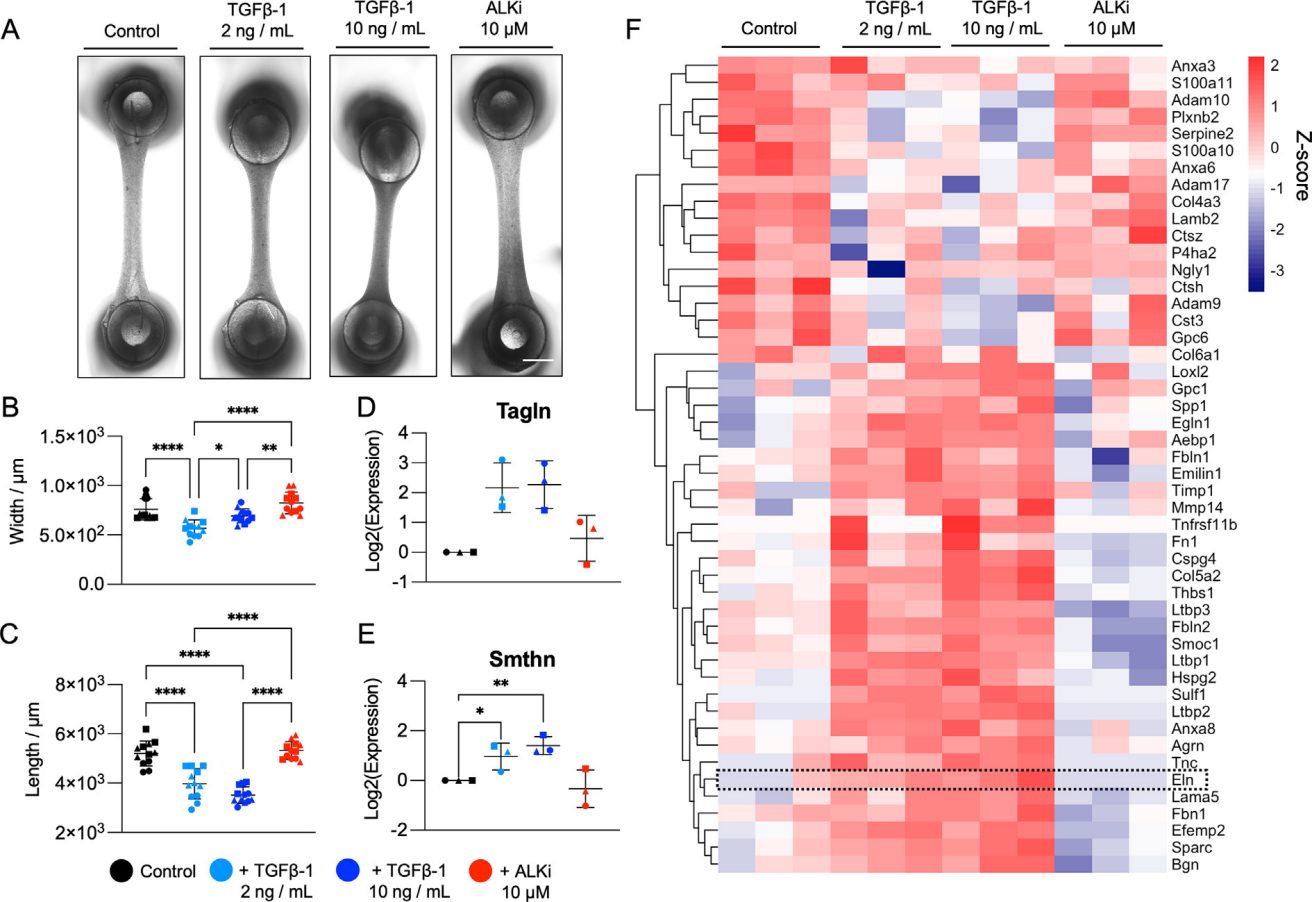
**TGFβ-1 stimulation of murine EVTs.** (**A**) Representative inverted light microscope images show changes in EVT construct length and width under control conditions or treatment with recombinant murine TGFβ-1 (2 or 10 ng / mL) or ALKi (SB431542) (10 μM) after 7 days of culture. Scale bar = 1 mm. Quantification of EVT width (**B**) and length (**C**) after 7 days of treatment under control conditions or treatment with TGFβ-1 (2 or 10 ng / mL) or ALKi (10 μM). N = 3 biological replicates, N = 3–4 technical replicates. Significance was determined by one-way ANOVA with Dunnett's multiple testing, a p value < 0.05 was considered significant. Shapes (circle, square, triangle) indicate independent SMC isolates. qPCR analysis of EVT SMC contractile marker expression transgelin (Tagln) (**D**) and smoothelin (Smthn) (**E**) after 7 days of treatment with TGFβ-1 (2 or 10 ng / mL) or ALKi. Shapes (circle, square, triangle) indicate independent SMC isolates. Significance was determined by one-way ANOVA with Dunnett’s correction for multiple comparisons. A p value < 0.05 was considered significant. (**F**) Heatmap of significantly changing ECM proteins after 7 days of treatment using Z-scores from log2 transformed protein abundances. Clustered heatmaps were generated from protein abundances using the pheatmap R package version 1.0.12. N = 3 biological replicates presented as an average of N = 2 technical replicates. Significance relative to the control group was determined using an unpaired Student’s t-test with a p value < 0.05 considered significant. Elastin (Eln) is highlighted.

TGFβ-1 signalling contributes to the maturation of SMC phenotype [37], stimulates ECM secretion [38] and can be blocked by treatment with inhibitors of activin receptor-like kinases (ALKi) [39]. To investigate the influence of TGFβ-1 on the physical remodelling of murine EVTs, inverted light microscope images were taken after EVTs were treated with recombinant TGFβ-1 (concentrations of 2 ng / mL and 10 ng / mL) or the ALKi SB 431542 (10 μM) for 7 days (Fig. 4**A**). Quantification of EVT construct length and width revealed EVTs became significantly shorter and thinner in response to TGFβ-1 relative to control untreated EVTs (**4B**-**C**). EVTs treated with TGFβ-1 demonstrated a dose-dependent increase in expression of the prototypical SMC contractile markers transgelin (Tagln) and smoothelin (Smthn) [40] (Fig. 4**D-E**).

### ECM remodelling in EVTs in response to TGFβ-1 stimulation

EVT lysates treated with TGFβ-1 (10 ng / mL) were found to have a significantly increased protein content compared to control groups (Supplementary Fig.6). Proteomics analysis revealed distinct ECM profiles after treatment with the different stimuli (Fig. 4**F**). Proteins significantly upregulated in TGFβ-1 (10 ng / mL) treated EVTs included collagens (Col6a1, Col5a2), elastin-binding proteins (Emilin1, Efemp2) and elastin (Eln). ALKi treated EVTs demonstrated the most similarity to the control group. Proteins that were significantly reduced after ALKi treatment included latent TGFβ binding protein (LTBP) family members LTBP-1 and -3.

As well as detecting ECM proteins, the proteomics method facilitated the detection of ECM protein receptors. ECM ligands and receptors identified in the treated EVTs were mapped using a manually curated murine ligand-receptor database: CellTalkDB [41]. Ligand-receptor pairs were filtered based on a significant change in abundance of one member of the pair after treatment with either TGFβ-1 (2 or 10 ng / mL), or ALKi (10 μM) (Supplementary Fig.7). As expected, the higher concentration of TGFβ-1 (10 ng / mL) induced the most changes with 23 ECM ligands found to interact with 21 receptor proteins. Receptors significantly affected by TGFβ-1 (10 ng / mL) stimulation included integrin alpha-2 (Itga2) and integrin beta-3 (Itgb3). In contrast to being upregulated after TGFβ-1 (10 ng / mL) treatment, the ligand-receptor pair fibrillin 1 (fbn1) - integrin alpha-V (Itgav) was significantly downregulated after ALKi treatment: Fbn1 mutations are the main cause for Marfan Syndrome.

### EVTs as a 3D model for vascular calcification

**Fig. 5:**
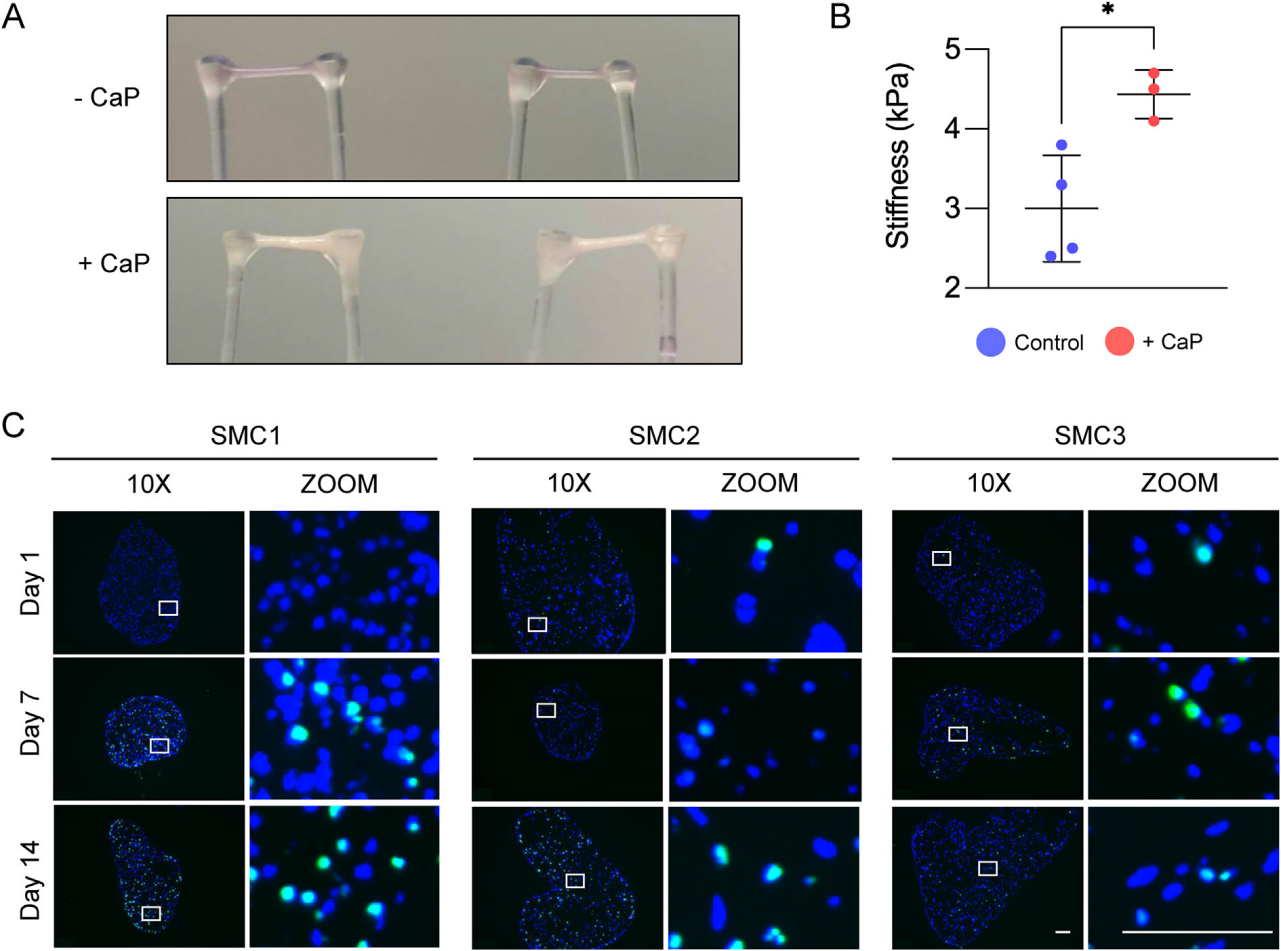
**Murine EVTs under calcifying conditions.** (**A**) EVTs cultured under control (-CaP) conditions are transparent. EVTs appear opaque and bone-like after 7 days of culture in pro-calcifying media (+CaP). (**B**) *Ex vivo* tensile testing measurements of calcified EVTs under control and pro-calcifying conditions after 7 days of culture. Shapes (circle, square, triangle) indicate independent SMC isolates. N = 3–4 technical replicates. Significance was determined by an unpaired Student’s t-test with a p value < 0.05 considered significant. (**C**) EVTs were cultured for 1, 7 or 14 days in low serum - CaP medium (5 % FBS), after which the EVTs were fixed, OCT-embedded and sliced to 10 μm thickness. TUNEL assay with counterstaining for DAPI was then performed. SMC1, SMC2 and SMC3 refers to independent aortic SMC isolates from different mice. Images show N = 3 biological replicates with TUNEL + cells in green and DAPI stained nuclei in blue. Scale bar = 100 μm.

**Fig. 6:**
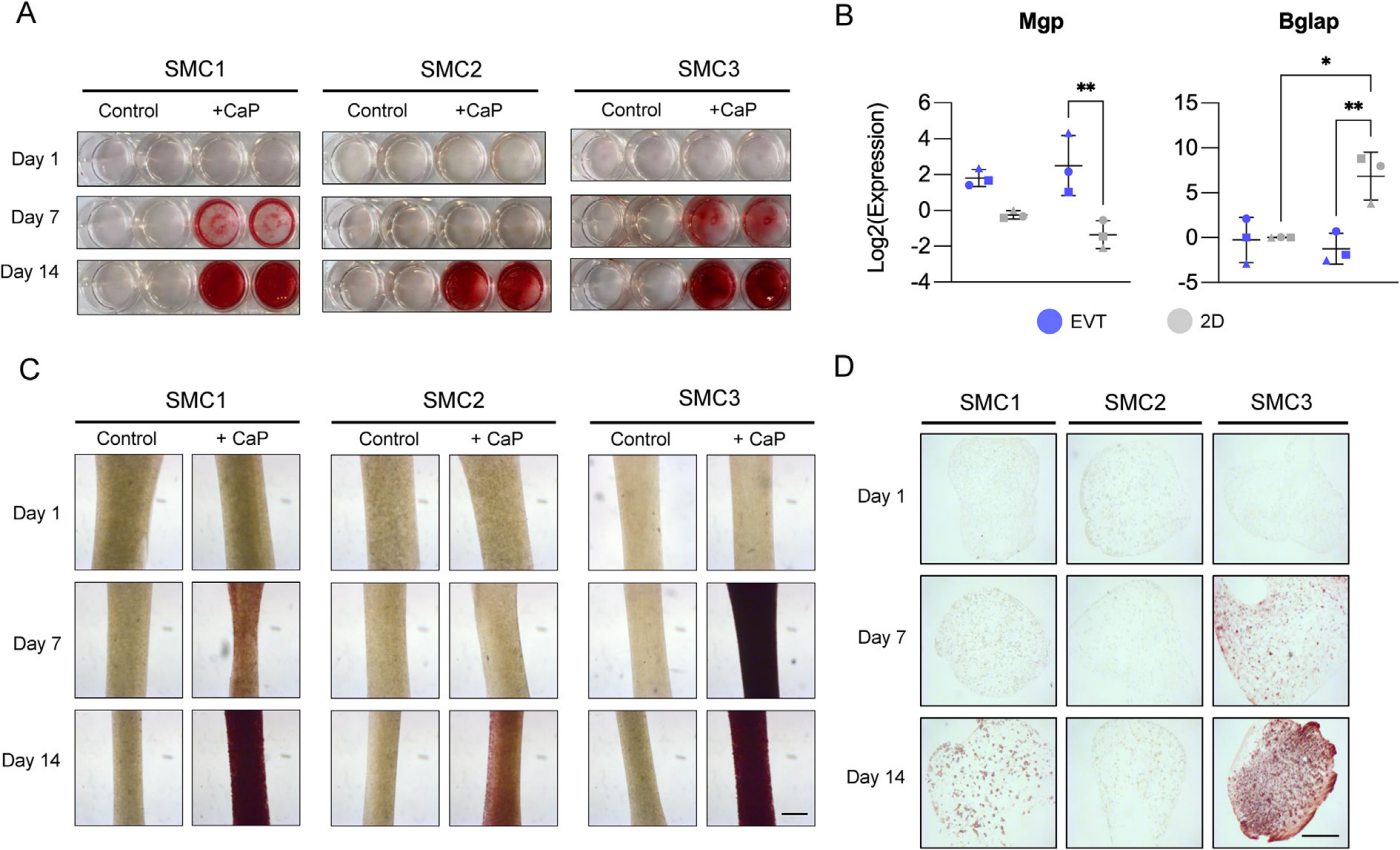
**Vascular calcification in murine EVTs.** (**A**) Alizarin Red S staining of 2D SMCs cultured under control or pro-calcifying conditions for 1, 7 or 14 days. (**B**) Transcript expression of matrix Gla protein (Mgp) and osteocalcin (Bglap) in EVTs and 2D SMCs cultured for 7 days under control or pro-calcifying conditions. N = 3 biological replicates presented as an average of 3–4 technical replicates. Error bars show SD. Significance was determined by two-way ANOVA with Šídák's multiple comparison test. A p value of < 0.05 was considered significant. (**C**) Alizarin Red S staining of whole mount EVTs cultured under control or pro-calcifying conditions for 1, 7 or 14 days. (**D**) Alizarin Red S staining of EVT transverse sections. N = 3 biological replicates and N = 2 technical replicates, per time point and condition. SMC1, SMC2 and SMC3 refers to independent aortic SMC isolates from different mice. Scale bars = 100 μm.

SMC calcification is associated with detrimental vascular pathologies including atherosclerosis [42]. EVTs became opaque and bone-like in appearance after 7 days of culture in high calcium phosphate medium (Fig. 5**A**). Calcified EVTs displayed significantly increased material stiffness as determined by *ex vivo* tensile testing measurements (Fig. 5**B**). Apoptotic SMCs within the ECM serve as a nidus for calcium nodule formation in the vasculature [43]. While apoptotic or necrotic SMCs tend to detach from the culture dish in conventional 2D cultures, TUNEL-positive SMCs were retained in EVTs cultured under pro-calcifying conditions (Fig. 5**C**). In 2D SMC cultures calcification is variable but once initiated it progresses rapidly until it is difficult to discern differences in the calcification properties between biological replicates at day 14 (Fig. 6**A**). A downregulation of the calcification inhibitor matrix Gla protein (Mgp) and upregulation of osteocalcin (Bglap), which strongly binds to apatite and calcium [44], in 2D SMCs may explain this finding (Fig. 6**B**). Strikingly, EVTs maintained higher Mgp and lower Bglap expression under calcifying conditions. Using the same primary SMCs from 3 different donor mice, the calcification response was more refined in EVTs compared to 2D cultures (Fig. 6**C and 6D**). Alizarin Red S staining of EVT transverse sections indicate that early calcification was initiated at the cellular level rather than extracellularly. However, upon extensive calcification the entire EVT construct (i.e., cells and ECM) was calcified throughout.

## Discussion

SMCs and their secreted ECM play a critical role in vessel homeostasis and vascular pathology. In the present study, we describe a 3D EVT model for studying ECM remodelling by SMCs in the context of vascular diseases. In addition to characterising EVTs under baseline culture conditions and comparing them to 2D controls, we implemented two applications for EVTs: treatment of EVTs with TGFβ-1 to induce contraction and alter their ECM composition as well as treatment with high calcium phosphate medium to recreate a disease-relevant vascular phenotype *in vitro*.

### Remodelling of EVTs

Immunohistochemistry revealed SMCs were oriented between the two PDMS posts. EVTs therefore displayed a tissue-like architecture where SMCs were aligned and maintained in a 3D environment surrounded by a functional and dynamic ECM. The addition of aprotinin to the EVT culture media slowed degradation of the fibrin matrix [45], however, substantial thinning of the EVT construct was observed over time. This finding was consistent in EVTs generated with both human and murine primary SMCs. SMCs cultured within EVTs downregulated expression of prototypical contractile markers. However, supplementation of the EVT media with TGFβ-1 induced SMC marker expression and ECM secretion. Furthermore, length and width measurements demonstrated that TGFβ-1 treatment impacts EVT dimensions by affecting construct contraction and thinning. We suspect the former is due to induction of a more contractile SMC phenotype, while the latter is likely to be a result of increased proteolytic activity [34]. For example, collagen-degrading matrix metalloproteinase 14 (Mmp14) [46] was significantly enriched in TGFβ-1 stimulated EVTs. Length and width remodelling in EVTs in response to pharmacological stimulation lends this model to applications that explore responses to drugs targeting SMC contractility [33], [47].

### Proteomics of EVTs

Typically, research in the tissue engineering field tends to focus on investigating individual proteins rather than adopting an unbiased and hypothesis-generating -*omics* approach [48]. Our laboratory has pioneered novel proteomics workflows to comprehensively interrogate the vascular extracellular proteome [49]. In 2011 we identified 126 core ECM proteins in human abdominal aortic aneurysm samples [50]. In 2017, Suna et al. identified a total of 151 ECM and ECM-associated proteins in porcine coronary arteries [51], whereas Langley et al. identified 136 ECM proteins in human carotid endarterectomies [52]. The identification of 135 core ECM proteins in EVTs is therefore comparable to our previous publications using mammalian vessels. Crucially, the ECM components found within EVTs are representative of the diverse ECM observed *in vivo*. By employing discovery proteomics alongside immunohistochemistry validation, our study provides the first comprehensive description of the extracellular proteome generated in 3D SMC tissues. Our findings provide insight into SMC adaptation to the 3D EVT environment. At day 1, upregulation of Serpin E1 (Plasminogen activator inhibitor 1) in EVTs may indicate a cellular response to the fibrin matrix [53]. Similarly, upregulation of inflammatory and injury related proteins including, Mac2 / Lgals3 [7], and Adam9 [54] in EVTs at this early time point may be conducive with an early inflammatory response. Among ECM proteins, the deposition of proteoglycans after 7 days of culture was most prominent, followed by the accumulation of ECM collagens and basement membrane proteins by day 14. This is consistent with a fibroblastic wound healing response and the evolution of a provisional ECM [55].

### Proteoglycans in EVTs

Retention of ECM components in fibrin hydrogels have previously been described. For example, decorin (Dcn) secreted by SMCs is retained within fibrin gels and mediates tissue contraction [56]. Through the employment of our previously published ECM proteomics methodology [49], we demonstrate that after prolonged culture the ECM of EVTs was dominated by accumulation of proteoglycans including a significant enrichment for Vcan, Bgn and Ogn; the latter of which, Ogn, was only detected after 14 days of EVT culture. Intriguingly, upregulation of proteoglycans is a hallmark of atherosclerosis development due to their ability to retain charged lipoprotein molecules in the early stages of the disease [57]. Corroborating this finding, Vcan and Bgn were found to co-localise with apolipoproteins in atherosclerotic plaques [24], [58]. Immunohistochemical analysis of EVT longitudinal sections using antibodies raised against full-length Vcan or its ADAMTS-cleaved product, versikine, were found in abundance in EVTs compared to the 2D pericellular matrix. Therefore, EVTs may be suited to study the proteoglycan turnover by their specific proteases *in vitro* [36], [49], [51], [59].

### Elastin in EVTs

SMC-secreted Eln is a major constituent of the arterial wall and an important contributor to vessel mechanics [14] and biochemistry [60]. The ability to invoke Eln synthesis *in vitro* within engineered SMC tissues is therefore considered an essential component to recreating the native physical and molecular SMC environment [61]. Although Eln was not detected within EVTs cultured under control conditions, significant upregulation of Eln was observed in the TGFβ-1 treated EVTs (**Supplementary Tables 1 and 2**). After treatment with TGFβ-1 for 7 days Eln was detected across all samples by mass spectrometry. Previously, SMCs embedded in fibrin gels and supplemented with TGFβ-1 (2.5 ng / mL) and insulin (2 μg / mL) for up to 5 weeks enhanced collagen and Eln secretion in parallel to displaying increased mechanical strength relative to untreated controls [62]. However, to our knowledge, EVTs are unique by enabling Eln expression in a 3D culture model after as early as 7 days of culture. As well as detecting Eln, a network of Eln-associated proteins including Eln binders (e.g. Emilin1 and Efemp2) [60] and post-translational regulators (e.g., Loxl2) [63] were also identified within EVTs. Furthermore, in line with the previous literature [64], induction of Eln synthesis within TGFβ-1 treated EVTs was associated with the engagement of SMC contractile fibers (i.e., upregulation of SMC contractile markers and shortening of EVT construct length). A physiologically relevant concentration of 2 ng / mL of TGFβ-1 was sufficient to mediate this effect [65].

### Cell-ECM interactions in EVTs

The proteomics approach employed enabled the identification of cell receptors alongside ECM proteins. Recently, Schuler et al. demonstrated using a proteomics approach how altered niche-cell communication in aged muscle stem cells in 2D *in vitro* culture contributes to altered cell functionality including reduced regenerative capacity [66]. Retention of ECM within EVTs facilitates a more physiological environment for investigation of SMC interactions with their extracellular niche than traditional 2D cultures [67]. We compared protein changes with TGFβ-1 by performing a parallel investigation of ligand-receptor pairs affected by activin-like kinase inhibition. As expected, inhibition resulted in opposite patterns to TGFβ-1 stimulation. Thus, proteomic analysis of EVTs can provide insight into how SMCs interact with their extracellular environment.

### EVTs for vascular disease modelling

Finally, we hypothesised that EVTs could be a novel and informative model for investigating vascular calcification due to i) accumulation of ECM; ii) loss of SMC contractile phenotype which is a prerequisite for SMC osteochondrogenesis [68]; iii) identification of calcification-related and calcium-binding proteins within EVTs. Unlike in 2D cultures, apoptotic cells were retained within EVTs and could contribute to the initiation of calcification [69]. On the addition of calcifying media EVTs demonstrated significantly increased material stiffness. A hallmark of age-associated arterial stiffness is the development of medial microcalcifications [70]. Therefore, EVTs can recapitulate features of *in vivo* vascular calcification. Thus far, we have used murine SMCs for calcification, but future studies will include human EVTs. The propensity of SMCs to calcify could be better distinguished in EVTs than 2D controls. Therefore, EVTs may be a more refined and sensitive model for detecting the onset and extent of calcification between different primary SMC donors.

### Limitations

EVTs are relatively high throughput compared to previously described 3D cell culture techniques [32], [33], [62]. However, the large number of cells required for EVT formation compared to conventional 2D cultures is a limitation; and higher passages of cells are needed to achieve adequate numbers of cells for biological and technical replicates. Future utilisation of human induced pluripotent stem cell-derived SMCs may help to alleviate this limitation associated with primary cell culture [71]. Technical limitations of a TUNEL assay include proliferating cells with increased rates of DNA repair may exhibit false TUNEL positivity [72]. In EVTs, however, we observed cell loss. Besides apoptosis, necrosis may cause ‘nonapoptotic’ DNA fragmentation. However, for the purpose of this experiment the TUNEL assay demonstrates retention of apopotic/necrotic SMCs within the 3D matrix of EVTs. For tensile testing, EVTs were assumed to have nominally identical dimensions following culture allowing for comparison between groups and relative stiffness to be compared. The calculated absolute values are within the range of other published data [29], however, to formally quantify the Young’s modulus monitoring the geometrical deformation of the specimen in non-axial planes during testing would be required.

### Future directions

Future adaptations of the model may include introduction of pulsatile stimulation to mimic physiological events. Additional cell read-outs such as cell proliferation, motility and metabolism could be investigated using the EVT model in the future. Demonstration of Eln synthesis within EVTs lends this model to future investigation into the regulation of tissue mechanical properties and ECM fiber alignment and maturation.

### Conclusion

SMCs within EVTs are aligned and secretory. In contrast to standard 2D cultures the nascent ECM is retained within EVTs facilitating 3D cell–cell and cell-ECM interactions. We propose EVTs as a novel, versatile and reproducible model for investigating SMC-ECM interactions. As well as being a potential model for pre-clinical drug screening, EVTs may be useful for investigating the impact of risk factors and genetic variants on SMCs.

## Experimental procedures

Further details regarding general reagents, commercial kits, enzymes, oligonucleotide primers, antibodies and SMC donors are provided in the Data Supplement (**Supplementary Tables 4 and 5**).

### Primary human and mouse SMC cultures

Human SMCs from adult donors were purchased from suppliers, Lonza or PromoCell. Wild-type SMCs were derived from Adamts5^ΔCat^ mice backcrossed onto a C57BL/6J background at least 10 times (i.e., >99.9 % wild-type C57BL/6J). Human and murine SMCs were maintained in DMEM basal media supplemented with 10 % FBS and 1 % glutamine, 1 % Penicillin-Streptomycin (Pen-Strep). For 2D comparisons SMCs were seeded onto uncoated tissue culture plastic at a density of 100,000 cells / well for a 12-well plate and 200,000 cells / well for a 6-well plate and cultured in EVT medium as described below. Human SMCs were used at an average of passage 10, murine SMCs were used at an average of passage 15. SMC isolates were always used within three passages of each other for comparability within the same experiment.

### Generation of EVTs

EVTs were casted using the adapted protocol for the formation of EHTs [34]. On the day of EVT generation, the EVT apparatus was washed thoroughly with Ultrapure Milli-Q® water (Milli-Q® H_2_O). The EVT apparatus and a 1 % agarose solution in PBS were sterilised in a bench top autoclave at 121 °C for 15 mins with a cooling cycle. Meanwhile, SMCs were trypsinised, resuspended in complete DMEM and counted using a Neubauer chamber. After centrifugation, cell pellets (5 × 10^5^ cells / EVT) were carefully resuspended in a master mix consisting of bovine fibrinogen solution (2.5 μL), 2X DMEM (6.05 μL) and basal medium (101.2 μL) per single EVT. Master mix volumes were calculated to allow for a 10 % pipetting error and could be multiplied based on the desired number of EVTs. To form the agarose moulds, 1.6 mL / well of sterile liquid agarose solution was pipetted into wells of a 24-well culture plate. Teflon spacers were placed immediately into the agarose containing wells and left for 10 min at room temperature (RT) to allow for the agarose to set. Once set, the Teflon spacers were carefully removed. The PDMS racks were then evenly positioned within the agarose moulds.

The SMC-master mix (97 μL) was combined with thrombin (3 μL, 100 U / mL) and quickly pipetted into an agarose mould between the PDMS racks. This process was repeated based on the number of EVTs to be formed. The 24-well plate was then incubated (37 °C, 5 % CO_2_, 1 hr) to allow for fibrin polymerisation and for the EVTs to set. Pre-warmed DMEM (500 μL) was carefully pipetted into each EVT-containing well and the plate was returned to the incubator for a further 15 min. The PDMS racks and suspended EVTs were then carefully lifted from the agarose moulds and placed into a new 24-well plate with 1.5 mL of pre-warmed EVT medium / well. EVT medium was: DMEM supplemented with 10 % FBS, 1 % l-Glutamine, 1 % Pen-Strep and bovine aprotinin (33 μg / mL). EVTs were cultured for up to 14 days before harvesting for biomolecular or histochemical analysis.

### EVT tissue fixation, storage and immunohistochemistry

EVTs were washed in pre-warmed PBS (1.5 mL / well) for 3 × 5 min. EVTs suspended on the PDMS racks were transferred into ice-cold 10 % neutral buffered formalin and stored overnight under gentle shaking (4 °C, 350 rpm). EVTs were then washed in ice-cold PBS for 3 × 5 min, removed from the PDMS racks using sterile tweezers and stored in 1.5 mL tubes containing 70 % ethanol at 4 °C. Paraffin-embedding, longitudinal sectioning (4 μm slices) and immunohistochemistry was performed as previously described [73]. Samples were stained according to standard procedures for hematoxylin & eosin (H&E), Alcian blue, Picrosirius red and Elastin van Gieson (EVG).

### Treatment of EVTs

Murine SMC EVTs were cultured in EVT medium supplemented with recombinant mouse transforming growth factor beta-1 (TGFβ-1) (10 ng / mL or 2 ng / mL) or the activin-like kinase (ALKi) inhibitor, SB 431542 (10 μM). Negative controls were cultured in EVT medium only. EVTs were harvested for protein and RNA isolation after 7 days of culture.

### Phase contrast images of EVTs

Transmission microscopy images of EVTs *in situ* were gathered using a Nikon Eclipse Ti-E microscope equipped with a LED light source, CFI Plan Achro 2x / 0.06NA objective lens and Nikon DS-Fi3 RGB digital camera using NIS-Elements software version 5.21 (Nikon).

### Protein extraction from EVT and 2D lysates

EVTs were washed in pre-warmed PBS (1.5 mL / well) for 3 × 5 min. EVTs were then removed from the PDMS racks using sterile tweezers and transferred into 2.0 mL tubes containing 200 μL of ice-cold cell lysis buffer with added proteinase and phosphatase inhibitors, and Lysing Matrix D beads. 2D SMCs were lysed according to the same protocol. Samples were homogenised for 4 × 30 sec cycles using a bench-top tissue homogeniser and kept cool on ice in between cycles. Cell suspensions were then centrifuged (10 min, 10,000 xg, 4 °C). Cell pellets were discarded, and supernatants were stored at −80 °C. A BCA assay was performed according to the manufacturer’s protocol to determine protein concentrations.

### Proteomics analysis of EVTs and 2D SMCs

15 μg of protein were precipitated in ethanol and deglycosylated using the following enzymes: chondroitinase (1:100), heparinase (1:500), keratanase (1:500), β1,4-galactosidase (1:200), β-*N*-acetylglucosaminidase (1:200), α2-3,6,8,9-neuraminidase (1:200), and O-glycosidase (1:200). Proteins were incubated at 25 °C for 2 hr and then 37 °C for 24 hr under shaking. Next, samples were dried in a vacuum concentrator (Thermo Scientific, Savant SpeedVac SPD131DDA) and resuspended in 15 μL ^18^O water + PNGase F (1:100) and incubated (37 °C, 48 hr) under agitation. Each sample was denatured with the addition of urea (6 M) and thiourea (2 M), then reduced by addition of dithiothreitol (10 mM) and incubated (37 °C, 1 hr). Iodoacetamide (50 mM) was added, and samples were incubated in the dark (RT, 1 hr). Proteins were then precipitated in ice-cold acetone (-20 °C, overnight). Samples were centrifuged (15000 × g, 35 min at 0 °C) and acetone was removed. Protein pellets were dried using a vacuum concentrator, resuspended in triethylammonium bicarbonate (0.1 M, pH 8.2) containing 1:50 trypsin:protein, and digested overnight (37 °C). The reaction was stopped by acidification with trifluoroacetic acid (TFA) (1 %). Peptide clean-up was achieved using a Bravo AssayMAP Liquid Handling Platform (Agilent). After conditioning and equilibration of the resin, acidified peptide solutions were loaded onto AssayMAP C18 Cartridges (Agilent, 5190–6532), washed using 1 % acetonitrile (ACN), 0.1 % TFA (aq) and eluted using 70 % ACN, 0.1 % TFA (aq). Eluted peptides were dried in a vacuum concentrator and resuspended at a concentration of 0.5 μg / μL using 2 % ACN, 0.05 % TFA (aq). Peptides were analysed using an UltiMate 3000 liquid chromatography (LC) system which was coupled via an EASY-Spray Source to a Q Exactive HF mass spectrometer (all Thermo Scientific). Peptides were injected onto a C18 trap cartridge (Thermo Scientific, 160454) at a flow rate of 25 μL / min for 1 min, using 0.1 % formic acid (FA, aq). Peptides were eluted from the trap cartridge and separated on an analytical column (Thermo Scientific, ES803A, at 45 °C) at a flow rate of 0.25 μL / min using the following gradient: 0–1 min, 1 % B; 1–6 min, 1–6 % B; 6–40 min, 6–18 % B; 40–70 min, 18–35 % B; 70–80 min, 35–45 % B; 80–81 min, 45–99 % B; 81–90 min, 99 % B; 90–120 min, 4 % B. Mobile phase A was 0.1 % FA (aq) and mobile phase B was 80 % ACN, 0.1 % FA (aq). Precursor spectra were acquired using Orbitrap detection (resolution 60,000 at 200 *m*/*z*, scan range 350–1600 *m*/*z*). Data-dependent fragment spectra of the most abundant precursor ions were obtained after higher-energy C-trap dissociation and Orbitrap detection (resolution 15,000 at 200 *m*/*z*) with TopN mode (loop count 15) and dynamic exclusion (duration 40 s) enabled.

### Database search of LC-MS/MS data and data filtering

The liquid chromatography tandem mass spectrometry (LC-MS/MS) files were processed using Proteome Discoverer (Thermo Scientific, version 2.3.0.523) and Mascot (Matrix Science, version 2.6.0). RAW files were searched against mouse databases: UniProtKB/Swiss-Prot version from January 2020, 17,033 protein entries or UniProtKB/Swiss-Prot, version from February 2021, 17,063 protein entries, and a custom-built foetal bovine serum database (249 protein entries). The mass tolerance was set at 10 ppm for precursor ions and 0.02 Da for fragment ions. Trypsin was set as the protein-digesting enzyme with up to two missed cleavages being allowed. Cysteine carbamidomethylation (static), oxidation of lysine/methionine/proline (dynamic), and deamidation of asparagine in presence of ^18^O water (dynamic) were chosen as modifications. The quality of peptide-spectrum-matches (PSMs) obtained from the Mascot target/decoy search was assessed using Percolator. PSM and peptide validation was done by Peptide Validator, where the target false discovery rate of PSMs and peptides was set at 0.01. Quantification was based on precursor intensity. Precursor abundances in all samples were normalised to the total peptide amount. Before exporting the data from Proteome Discoverer for further analysis, it was filtered to contain Master Proteins only, and a minimum number of one unique peptide per protein.

### Western blotting

Protein samples (10–20 μg) were denatured in sample buffer (0.1 M Tris, pH 6.8, 40 % glycerol, 2 % SDS, 2 % beta-mercaptoethanol and 0.02 % bromphenol blue) for 5 mins, 95 °C. Samples were loaded onto NuPAGE 4–12 % Bis Tris Gels and ran at 150 V. A MagicMarkTM XP Western Protein Standard was used as a molecular weight reference. Proteins were transferred onto nitrocellulose membranes at 350 mA for 2 hr and blocked in fat-free milk powder (5 %) diluted in PBS-Tween (RT, 1 hr). Membranes were incubated with primary antibodies diluted in PBS-Tween with 5 % BSA and 0.02 % sodium azide overnight at 4 °C. The next day, membranes were incubated with the appropriate secondary horseradish peroxidase (HRP)-conjugated secondary antibodies diluted in 5 % milk powder in PBS-TWEEN (RT, 1 hr). Blots were imaged using ECL Western Blotting Detection Reagent on Medical X-ray film and developed using an X-ray film processor.

### RNA extraction from EVTs and 2D SMCs

EVTs were washed in pre-warmed PBS (1.5 mL / well) for 3 × 5 min. EVTs were then removed from the PDMS racks using sterile tweezers and placed into 1.5 mL Eppendorf® Tubes containing 700 μL Qiazol® Lysis Reagent. In parallel, 2D SMCs were washed 3x with pre-warmed PBS. 700 μL Qiazol® was added to each well and cells were collected by rigorous pipetting and scraping before transfer into new 1.5 mL Eppendorf® Tubes. EVTs and 2D SMCs in Qiazol® were stored at −80 °C.

For RNA extraction the SMC-Qiazol® tubes were thawed on ice. Proteinase K (100 μg / mL) was added to each EVT-containing tube before incubation under shaking (55 °C, 15 min, 650 rpm). The EVT-Qiazol® solution was then triturated utilising a p200 micropipette until the EVT dissociated into smaller pieces. 2D SMCs in Qiazol® did not require this step and were kept on ice. RNA was extracted from EVTs and 2D controls utilising the miRNeasy Mini Kit, according to the manufacturer’s instructions. RNA quality and concentration was determined using a Nanodrop® spectrophotometer.

### cDNA synthesis and RT-qPCR

cDNA was synthesised using the SuperScriptTM VILO^TM^ cDNA Synthesis Kit on the Veriti thermal cycler. RT-qPCR was carried out using the SYBR Green PCR master mix and RNase free water in a ViiA 7 RT-qPCR system. Each run consisted of 40 cycles of denaturation (30 s at 95 °C), annealing (30 s at 60 °C) and extension (15 s at 95 °C). The relative mRNA expression was determined using the comparative threshold cycle (Ct) method, assuming a 100 % amplification efficiency [74]. Murine primers were designed using Primer-BLAST [75]. The relative expression of the target gene was normalised to housekeeping gene, apoptosis inhibitor 5 (Api5) [76].

### Calcification assays

For calcification assays instead of using EVT medium, complete M199 medium containing 5 % FBS, 1 % Pen-strep, 1 % l-glutamine and aprotinin (33 μg / mL) was used. After 24 h in culture, calcification was induced by media supplementation with calcium (2.7 mM) and phosphate (2.5 mM), as previously described [51]. For non-calcifying controls, complete M199 medium was used with no additional calcium or phosphate (1.8 and 1 mM, respectively). EVT and 2D controls were cultured for up to 14 days under calcifying conditions.

### Tensile testing

The stiffness of the EVTs was estimated by tensile testing using a universal testing apparatus. EVTs were carefully demounted from the cell culture plasticware but remained attached at each end to the mounting posts. The EVT was aligned vertically and centred between two grips which were attached to the head of each mounting post so that the grip engaged close to but not in contact with the EVT. The mounting operation was consistently conducted within 5 mins to maintain the hydration of the EVT. Tensile loading was undertaken using a displacement control of 1 mm / min with force recorded at 50 ms intervals, using a calibrated and balanced 10 N load cell and was controlled by Bluehill software v 4.13. All measurements were performed at 23 ± 1 °C.

### Measurement of EVT stiffness

EVT stiffness was estimated by identifying the linear portion of the load displacement plots within the first 1.5 mm of scaffold extension. Linearity was confirmed by fitting a linear regression trendline with R^2^ > 0.99. Stiffness (k) was estimated as a function of force (F) and displacement (δ) (Hooke’s Law: k = Fδ), reported in kilopascals (kPa) and by assuming a constant cross-sectional area (within the first 1.5 mm of extension). An estimated stiffness is reported as opposed to a Young’s modulus calculation due to lack of measurement of Poission’s contraction during testing. Results were analysed in a randomised and blinded fashion.

### EVT OCT embedding and cryosectioning

Fixed EVTs were washed in PBS (1.5 mL / well) for 3 × 15 min. EVTs were then sequentially submerged in 1) 30 % sucrose in PBS overnight at 4 °C, 2) 50 % sucrose, 50 % OCT overnight at 4 °C, 3) 100 % OCT overnight at 4 °C in a 48-well format (200 μL / well). Samples were then placed into a cryomold containing OCT embedding matrix and rapidly frozen over 2-methylbutane on dry ice, before storage at −80 °C. Prior to sectioning, the OCT-embedded EVTs were equilibrated to the cryostat chamber temperature. Transverse EVT sections were sliced to a thickness of 10 μm and collected by adhering to a glass slide. Slides were stored at −20 °C.

### TUNEL staining

*In situ* cell death was detected by a terminal deoxynucleotidyl transferase dUTP Nick-End Labelling (TUNEL) assay on EVT transverse cryosections (10 μm), according to the manufacturer’s protocol. DAPI was used as a counterstain. Briefly, slides were thawed (RT, 10 mins). Sections were fixed for 10 mins in freshly made 4 % PFA, before washing in PBS. Sections were permeabilised in 0.1 % Triton X-100 for 15 mins and washed with PBS. Positive controls were treated with 30 U / mL DNase II (15 mins, 37 °C) prior to TUNEL staining. Samples were incubated with the manufacturer’s TUNEL reaction mixture consisting of 90 % label solution and 10 % enzyme solution and incubated in a humidified chamber (37 °C, 1 hr). Negative controls were treated the same, but with the label solution only. Slides were washed with PBS and nuclei were counterstained with DAPI (1:5000) (10 mins at RT). After washing and residual liquid removal, glass cover slips were mounted onto the slides with fluorescent mounting medium. Slides were imaged on the Digital Color Camera interfaced to LAS software.

### Alizarin Red S staining for calcification

A 0.5 % Alizarin Red S solution (pH 4.1) was freshly prepared. For whole mount EVT sections, EVTs were washed for 3 × 5 min in PBS and transferred to Eppendorf® tube’s containing the Alizarin Red S solution for 5 min. EVTs were then washed with MilliQ® H_2_O until the residual water was clear. Excess water was removed, and EVTs were placed into dented microscope slides followed by mounting media and a coverslip and allowed to set (24 hr, RT, in the dark). The same protocol was utilised for Alizarin Red S staining of EVT cryosections (10 μm), however, staining of slides was performed using a glass vertical staining jar. After washing and residual liquid removal, glass cover slips were mounted onto the slides with fluorescent mounting medium. Alizarin Red S stained EVT slides were imaged using the Digital Color Camera interfaced to LAS software. For Alizarin Red S staining of 2D calcified SMCs in a 24-well plate format SMCs were washed 3 × with PBS. SMCs were then fixed in 10 % paraformaldehyde for 10 mins before washing with PBS for a further 3 × 5 mins under gentle agitation (150 rpm). Fixed SMCs were then incubated with the 0.5 % Alizarin red S solution (30 min, RT). After gentle washing with PBS images of the plate were taken using an iPhone X camera phone (Apple Inc).

### Statistical methods

Statistical analyses were generated using GraphPad Prism version 9.0 for Mac, GraphPad Software, San Diego, California USA, www.graphpad.com. The appropriate tests were chosen according to the comparison as indicated in each figure legend. Data are represented as mean ± standard deviation (SD). A p value < 0.05 = *; <0.01 = **; <0.001 = ***; <0.0001 = ****. Proteomics data were filtered for proteins with < 70 % missing values across all samples, or 0 % missing values across one comparison group. For statistical analysis missing values were imputed. To interrogate the specific changes in the ECM, the LC-MS/MS data was filtered for ECM or ECM-related proteins, utilising the MatrisomeDB online annotator tool [77] and manual selection. Gene Ontology enrichment analysis was performed using the DAVID Bioinformatics Resource 6.8 [78], [79]. Cytoscape with StringApp plugin was used for network analysis and visualisation of proteomics data [80], [81]. Clustered heatmaps were generated from protein abundances using pheatmap R package version 1.0.12.

## Funding

E.R. is funded by a BHF PhD studentship (FS/17/65/33481). JBB is a BHF intermediate research fellow (FS/19/33/34328). F.C. was supported by funds from the DFG (CU 53/5-1), the Deutsche Stiftung für Herzforschung (F/57/20), the Werner-Otto-Stiftung (08/99) and the DZHK. M.M. is a British Heart Foundation (BHF) Chair Holder (CH/16/3/32406) with BHF programme grant support (RG/16/14/32397, RG/F/21/110053). M.M. received support from the BHF Centre for Vascular Regeneration with Edinburgh/Bristol (RM/17/3/33381) and from the excellence initiative (Competence Centers for Excellent Technologies – COMET) of the Austrian Research Promotion Agency FFG (K-Project No. 843536) funded by the BMVIT, BMWFW, Wirtschaftsagentur Wien, Wirtschafts- und Forschungsförderung Salzburg and Standortagentur Tirol. M.M. is also supported by the Leducq Foundation (18CVD02) and VASCage-C (Research Centre on Vascular Ageing and Stroke), an R&D K-Centre of the Austrian Research Promotion Agency (COMET program—Competence Centres for Excellent Technologies) funded by the Austrian Ministry for Transport, Innovation and Technology, the Austrian Ministry for Digital and Economic Affairs and the federal states Tyrol, Salzburg and Vienna with the grant number FSG 868624. We thank the Wohl Cellular Imaging Centre at King’s College London for help with light microscopy.

## CRediT authorship contribution statement

**Ella Reed:** Conceptualization, Methodology, Investigation, Writing – original draft, Formal analysis, Visualization. **Adam Fellows:** Conceptualization, Methodology, Investigation, Validation. **Ruifang Lu:** Investigation. **Marieke Rienks:** Writing – review & editing. **Lukas Schmidt:** Data curation, Resources. **Xiaoke Yin:** Data curation, Resources, Software. **Elisa Duregotti:** Writing – review & editing. **Mona Brandt:** Investigation. **Susanne Krasemann:** Investigation. **Kristin Hartmann:** Investigation. **Javier Barallobre-Barreiro:** Resources, Funding acquisition, Writing – review & editing. **Owen Addison:** Investigation, Methodology. **Friederike Cuello:** Conceptualization, Investigation, Writing – review & editing, Resources. **Arne Hansen:** Methodology, Writing – review & editing. **Manuel Mayr:** Conceptualization, Supervision, Funding acquisition, Project administration.

## Declaration of Competing Interest

The authors declare that they have no known competing financial interests or personal relationships that could have appeared to influence the work reported in this paper.

## References

[1] Libby P. (2021). The biology of atherosclerosis comes full circle: lessons for conquering cardiovascular disease. Nat. Rev. Cardiol..

[2] Stary H.C., Chandler A.B., Dinsmore R.E., Fuster V., Glagov S., Insull W., Rosenfeld M.E., Schwartz C.J., Wagner W.D., Wissler R.W. (1995). A Definition of Advanced Types of Atherosclerotic Lesions and a Histological Classification of Atherosclerosis: A Report From the Committee on Vascular Lesions of the Council on Arteriosclerosis, American Heart Association. ATVB.

[3] Boren J., Chapman M.J., Krauss R.M., Packard C.J., Bentzon J.F., Binder C.J., Daemen M.J., Demer L.L., Hegele R.A., Nicholls S.J. (2020). Low-density lipoproteins cause atherosclerotic cardiovascular disease: pathophysiological, genetic, and therapeutic insights: a consensus statement from the European Atherosclerosis Society Consensus Panel. Eur. Heart J..

[4] Nayor M., Brown K.J., Vasan R.S. (2021). The Molecular Basis of Predicting Atherosclerotic Cardiovascular Disease Risk. Circ. Res..

[5] Bennett M.R., Sinha S., Owens G.K. (2016). Vascular Smooth Muscle Cells in Atherosclerosis. Circ. Res..

[6] Shankman L.S., Gomez D., Cherepanova O.A., Salmon M., Alencar G.F., Haskins R.M., Swiatlowska P., Newman A.A.C., Greene E.S., Straub A.C., Isakson B., Randolph G.J., Owens G.K. (2015). KLF4-dependent phenotypic modulation of smooth muscle cells has a key role in atherosclerotic plaque pathogenesis. Nat. Med..

[7] Alencar G.F., Owsiany K.M., Karnewar S., Sukhavasi K., Mocci G., Nguyen A.T., Williams C.M., Shamsuzzaman S., Mokry M., Henderson C.A., Haskins R., Baylis R.A., Finn A.V., McNamara C.A., Zunder E.R., Venkata V., Pasterkamp G., Björkegren J., Bekiranov S., Owens G.K. (2020). Stem Cell Pluripotency Genes Klf4 and Oct4 Regulate Complex SMC Phenotypic Changes Critical in Late-Stage Atherosclerotic Lesion Pathogenesis. Circulation.

[8] Bentzon J.F., Majesky M.W. (2018). Lineage tracking of origin and fate of smooth muscle cells in atherosclerosis. Cardiovasc. Res..

[9] Pan H., Xue C., Auerbach B.J., Fan J., Bashore A.C., Cui J., Yang D.Y., Trignano S.B., Liu W., Shi J., Ihuegbu C.O., Bush E.C., Worley J., Vlahos L., Laise P., Solomon R.A., Connolly E.S., Califano A., Sims P.A., Zhang H., Li M., Reilly M.P. (2020). Single-Cell Genomics Reveals a Novel Cell State During Smooth Muscle Cell Phenotypic Switching and Potential Therapeutic Targets for Atherosclerosis in Mouse and Human. Circulation.

[10] Dobnikar L., Taylor A.L., Chappell J., Oldach P., Harman J.L., Oerton E., Dzierzak E., Bennett M.R., Spivakov M., Jorgensen H.F. (2018). Disease-relevant transcriptional signatures identified in individual smooth muscle cells from healthy mouse vessels. Nat. Commun..

[11] Madan M., Bishayi B., Hoge M., Amar S. (2008). Atheroprotective role of interleukin-6 in diet- and/or pathogen-associated atherosclerosis using an ApoE heterozygote murine model. Atherosclerosis..

[12] Gomez D., Owens G.K. (2012). Smooth muscle cell phenotypic switching in atherosclerosis. Cardiovasc. Res..

[13] Hynes R.O., Naba A. (2012). Overview of the matrisome–an inventory of extracellular matrix constituents and functions. Cold Spring Harb Perspect. Biol..

[14] Chow M.J., Turcotte R., Lin C.P., Zhang Y. (2014). Arterial extracellular matrix: a mechanobiological study of the contributions and interactions of elastin and collagen. Biophys. J ..

[15] Adiguzel E., Ahmad P.J., Franco C., Bendeck M.P. (2009). Collagens in the progression and complications of atherosclerosis. Vasc Med..

[16] Iozzo R.V., Schaefer L. (2015). Proteoglycan form and function: A comprehensive nomenclature of proteoglycans. Matrix Biol..

[17] Koch C.D., Lee C.M., Apte S.S. (2020). Aggrecan in Cardiovascular Development and Disease. J. Histochem. Cytochem..

[18] Cikach F.S., Koch C.D., Mead T.J., Galatioto J., Willard B.B., Emerton K.B., Eagleton M.J., Blackstone E.H., Ramirez F., Roselli E.E. (2018). Massive aggrecan and versican accumulation in thoracic aortic aneurysm and dissection. JCI Insight..

[19] Kalamajski S., Oldberg A. (2010). The role of small leucine-rich proteoglycans in collagen fibrillogenesis. Matrix Biol..

[20] Schaefer L., Iozzo R.V. (2008). Biological functions of the small leucine-rich proteoglycans: from genetics to signal transduction. J. Biol. Chem..

[21] Lyck Hansen M., Beck H.C., Irmukhamedov A., Jensen P.S., Olsen M.H., Rasmussen L.M. (2015). Proteome analysis of human arterial tissue discloses associations between the vascular content of small leucine-rich repeat proteoglycans and pulse wave velocity. Arterioscler. Thromb. Vasc. Biol..

[22] Nakashima Y., Wight T.N., Sueishi K. (2008). Early atherosclerosis in humans: role of diffuse intimal thickening and extracellular matrix proteoglycans. Cardiovasc. Res..

[23] Talusan P., Bedri S., Yang S., Kattapuram T., Silva N., Roughley P.J., Stone J.R. (2005). Analysis of intimal proteoglycans in atherosclerosis-prone and atherosclerosis-resistant human arteries by mass spectrometry. Mol. Cell. Proteomics.

[24] Didangelos A., Mayr U., Monaco C., Mayr M. (2012). Novel role of ADAMTS-5 protein in proteoglycan turnover and lipoprotein retention in atherosclerosis. J. Biol. Chem..

[25] Liddy K.A., White M.Y., Cordwell S.J. (2013). Functional decorations: post-translational modifications and heart disease delineated by targeted proteomics. Genome Med..

[26] Mead T.J., Bhutada S., Martin D.R., Apte S.S. (2022). Proteolysis: a key post-translational modification regulating proteoglycans. Am. J. Physiol. Cell Physiol..

[27] Harman J.L., Jorgensen H.F. (2019). The role of smooth muscle cells in plaque stability: Therapeutic targeting potential. Br. J. Pharmacol..

[28] Lutolf M.P., Gilbert P.M., Blau H.M. (2009). Designing materials to direct stem-cell fate. Nature.

[29] Guimarães C.F., Gasperini L., Marques A.P., Reis R.L. (2020). The stiffness of living tissues and its implications for tissue engineering. Nat. Rev. Mater..

[30] Xie S.A., Zhang T., Wang J., Zhao F., Zhang Y.P., Yao W.J., Hur S.S., Yeh Y.T., Pang W., Zheng L.S. (2018). Matrix stiffness determines the phenotype of vascular smooth muscle cell in vitro and in vivo: Role of DNA methyltransferase 1. Biomaterials.

[31] Derricks K.E., Rich C.B., Buczek-Thomas J.A., Nugent M.A. (2013). Ascorbate enhances elastin synthesis in 3D tissue-engineered pulmonary fibroblasts constructs. Tissue Cell.

[32] L'Heureux N., Stoclet J.C., Auger F.A., Lagaud G.J., Germain L., Andriantsitohaina R. (2001). A human tissue-engineered vascular media: a new model for pharmacological studies of contractile responses. FASEB J..

[33] Roh J.D., Sawh-Martinez R., Brennan M.P., Jay S.M., Devine L., Rao D.A., Yi T., Mirensky T.L., Nalbandian A., Udelsman B., Hibino N., Shinoka T., Saltzman W.M., Snyder E., Kyriakides T.R., Pober J.S., Breuer C.K. (2010). Tissue-engineered vascular grafts transform into mature blood vessels via an inflammation-mediated process of vascular remodeling. Proc Natl Acad Sci U S A..

[34] Breckwoldt K., Letuffe-Brenière D., Mannhardt I., Schulze T., Ulmer B., Werner T., Benzin A., Klampe B., Reinsch M.C., Laufer S., Shibamiya A., Prondzynski M., Mearini G., Schade D., Fuchs S., Neuber C., Krämer E., Saleem U., Schulze M.L., Rodriguez M.L., Eschenhagen T., Hansen A. (2017). Differentiation of cardiomyocytes and generation of human engineered heart tissue. Nat. Protoc..

[35] Lindner V., Wang Q., Conley B.A., Friesel R.E., Vary C.P. (2005). Vascular injury induces expression of periostin: implications for vascular cell differentiation and migration. Arterioscler. Thromb. Vasc. Biol..

[36] Fava M., Barallobre-Barreiro J., Mayr U., Lu R., Didangelos A., Baig F., Lynch M., Catibog N., Joshi A., Barwari T., Yin X., Jahangiri M., Mayr M. (2018). Role of ADAMTS-5 in Aortic Dilatation and Extracellular Matrix Remodeling. Arterioscler. Thromb. Vasc. Biol..

[37] Tang Y., Yang X., Friesel R.E., Vary C.P., Liaw L. (2011). Mechanisms of TGF-beta-induced differentiation in human vascular smooth muscle cells. J. Vasc. Res..

[38] Lutgens E., Gijbels M., Smook M., Heeringa P., Gotwals P., Koteliansky V.E., Daemen M.J. (2002). Transforming growth factor-beta mediates balance between inflammation and fibrosis during plaque progression. Arterioscler. Thromb. Vasc. Biol..

[39] Inman G.J., Nicolas F.J., Callahan J.F., Harling J.D., Gaster L.M., Reith A.D., Laping N.J., Hill C.S. (2002). SB-431542 is a potent and specific inhibitor of transforming growth factor-beta superfamily type I activin receptor-like kinase (ALK) receptors ALK4, ALK5, and ALK7. Mol. Pharmacol..

[40] van Eys G.J., Niessen P.M., Rensen S.S. (2007). Smoothelin in vascular smooth muscle cells. Trends Cardiovasc. Med..

[41] Shao X., Liao J., Li C., Lu X., Cheng J., Fan X. (2021). Cell TalkDB: a manually curated database of ligand-receptor interactions in humans and mice. Brief Bioinform..

[42] Durham A.L., Speer M.Y., Scatena M., Giachelli C.M., Shanahan C.M. (2018). Role of smooth muscle cells in vascular calcification: implications in atherosclerosis and arterial stiffness. Cardiovasc. Res..

[43] Basalyga D.M., Simionescu D.T., Xiong W., Baxter B.T., Starcher B.C., Vyavahare N.R. (2004). Elastin degradation and calcification in an abdominal aorta injury model: role of matrix metalloproteinases. Circulation.

[44] Hauschka P.V., Wians F.H. (1989). Osteocalcin-hydroxyapatite interaction in the extracellular organic matrix of bone. Anat. Rec..

[45] Muhleder S., Pill K., Schaupper M., Labuda K., Priglinger E., Hofbauer P., Charwat V., Marx U., Redl H., Holnthoner W. (2018). The role of fibrinolysis inhibition in engineered vascular networks derived from endothelial cells and adipose-derived stem cells. Stem Cell Res. Ther..

[46] Lee H., Overall C.M., McCulloch C.A., Sodek J., Nusrat A. (2006). A critical role for the membrane-type 1 matrix metalloproteinase in collagen phagocytosis. Mol. Biol. Cell.

[47] Peng H.F., Liu J.Y., Andreadis S.T., Swartz D.D. (2011). Hair follicle-derived smooth muscle cells and small intestinal submucosa for engineering mechanically robust and vasoreactive vascular media. Tissue Eng. Part A.

[48] Joshi A., Rienks M., Theofilatos K., Mayr M. (2021). Systems biology in cardiovascular disease: a multiomics approach. Nat. Rev. Cardiol..

[49] Barallobre-Barreiro J., Baig F., Fava M., Yin X., Mayr M. (2017). Glycoproteomics of the Extracellular Matrix: A Method for Intact Glycopeptide Analysis Using Mass Spectrometry. J Vis Exp..

[50] Didangelos A., Yin X., Mandal K., Saje A., Smith A., Xu Q., Jahangiri M., Mayr M. (2011). Extracellular matrix composition and remodeling in human abdominal aortic aneurysms: a proteomics approach. Mol. Cell. Proteomics.

[51] Suna G., Wojakowski W., Lynch M., Barallobre-Barreiro J., Yin X., Mayr U., Baig F., Lu R., Fava M., Hayward R., Molenaar C., White S.J., Roleder T., Milewski K.P., Gasior P., Buszman P.P., Buszman P., Jahangiri M., Shanahan C.M., Hill J., Mayr M. (2018). Extracellular Matrix Proteomics Reveals Interplay of Aggrecan and Aggrecanases in Vascular Remodeling of Stented Coronary Arteries. Circulation.

[52] Langley S.R., Willeit K., Didangelos A., Matic L.P., Skroblin P., Barallobre-Barreiro J., Lengquist M., Rungger G., Kapustin A., Kedenko L. (2017). Extracellular matrix proteomics identifies molecular signature of symptomatic carotid plaques. J. Clin. Invest..

[53] Morrow G.B., Whyte C.S., Mutch N.J. (2021). A Serpin With a Finger in Many PAIs: PAI-1's Central Function in Thromboinflammation and Cardiovascular Disease. Front Cardiovasc Med..

[54] Shen G., Sun Q., Yao Y., Li S., Liu G., Yuan C., Li H., Xu Y., Wang H. (2020). Role of ADAM9 and miR-126 in the development of abdominal aortic aneurysm. Atherosclerosis..

[55] Barker T.H., Engler A.J. (2017). The provisional matrix: setting the stage for tissue repair outcomes. Matrix Biol..

[56] Johnson P.Y., Potter-Perigo S., Gooden M.D., Vernon R.B., Wight T.N. (2007). Decorin synthesized by arterial smooth muscle cells is retained in fibrin gels and modulates fibrin contraction. J. Cell. Biochem..

[57] Wight T.N., Merrilees M.J. (2004). Proteoglycans in atherosclerosis and restenosis: key roles for versican. Circ. Res..

[58] O'Brien K.D., Olin K.L., Alpers C.E., Chiu W., Ferguson M., Hudkins K., Wight T.N., Chait A. (1998). Comparison of apolipoprotein and proteoglycan deposits in human coronary atherosclerotic plaques: colocalization of biglycan with apolipoproteins. Circulation.

[59] Dupuis L.E., McCulloch D.R., McGarity J.D., Bahan A., Wessels A., Weber D., Diminich A.M., Nelson C.M., Apte S.S., Kern C.B. (2011). Altered versican cleavage in ADAMTS5 deficient mice; a novel etiology of myxomatous valve disease. Dev. Biol..

[60] Brooke B.S., Bayes-Genis A., Li D.Y. (2003). New insights into elastin and vascular disease. Trends Cardiovasc. Med..

[61] Patel A., Fine B., Sandig M., Mequanint K. (2006). Elastin biosynthesis: The missing link in tissue-engineered blood vessels. Cardiovasc. Res..

[62] Ross J.J., Tranquillo R.T. (2003). ECM gene expression correlates with in vitro tissue growth and development in fibrin gel remodeled by neonatal smooth muscle cells. Matrix Biol..

[63] Schmelzer C.E.H., Heinz A., Troilo H., Lockhart‐Cairns M.P., Jowitt T.A., Marchand M.F., Bidault L., Bignon M., Hedtke T., Barret A., McConnell J.C., Sherratt M.J., Germain S., Hulmes D.J.S., Baldock C., Muller L. (2019). Lysyl oxidase-like 2 (LOXL2)-mediated cross-linking of tropoelastin. FASEB J..

[64] Karnik S.K., Brooke B.S., Bayes-Genis A., Sorensen L., Wythe J.D., Schwartz R.S., Keating M.T., Li D.Y. (2003). A critical role for elastin signaling in vascular morphogenesis and disease. Development..

[65] Fogel-Petrovic M., Long J.A., Misso N.L., Foster P.S., Bhoola K.D., Thompson P.J. (2007). Physiological concentrations of transforming growth factor beta1 selectively inhibit human dendritic cell function. Int. Immunopharmacol..

[66] Schüler S.C., Kirkpatrick J.M., Schmidt M., Santinha D., Koch P., Di Sanzo S., Cirri E., Hemberg M., Ori A., von Maltzahn J. (2021). Extensive remodeling of the extracellular matrix during aging contributes to age-dependent impairments of muscle stem cell functionality. Cell Rep..

[67] Duval K., Grover H., Han L.H., Mou Y., Pegoraro A.F., Fredberg J., Chen Z. (2017). Modeling Physiological Events in 2D vs. 3D Cell Culture. Physiology (Bethesda).

[68] Speer M.Y., Yang H.Y., Brabb T., Leaf E., Look A., Lin W.L., Frutkin A., Dichek D., Giachelli C.M. (2009). Smooth muscle cells give rise to osteochondrogenic precursors and chondrocytes in calcifying arteries. Circ. Res..

[69] Clarke M.C., Littlewood T.D., Figg N., Maguire J.J., Davenport A.P., Goddard M., Bennett M.R. (2008). Chronic apoptosis of vascular smooth muscle cells accelerates atherosclerosis and promotes calcification and medial degeneration. Circ. Res..

[70] Mackey R.H., Venkitachalam L., Sutton-Tyrrell K. (2007). Calcifications, arterial stiffness and atherosclerosis. Adv. Cardiol..

[71] Dash B., Levi K., Schwan J., Luo J., Bartulos O., Wu H., Qiu C., Yi T., Ren Y., Campbell S., Rolle M., Qyang Y. (2016). Tissue-Engineered Vascular Rings from Human iPSC-Derived Smooth Muscle Cells. Stem Cell Rep..

[72] Liu K.X., Chen G.P., Lin P.L., Huang J.C., Lin X., Qi J.C., Lin Q.C. (2018). Detection and analysis of apoptosis- and autophagy-related miRNAs of mouse vascular endothelial cells in chronic intermittent hypoxia model. Life Sci..

[73] Mannhardt I., Breckwoldt K., Letuffe-Brenière D., Schaaf S., Schulz H., Neuber C., Benzin A., Werner T., Eder A., Schulze T., Klampe B., Christ T., Hirt M., Huebner N., Moretti A., Eschenhagen T., Hansen A. (2016). Human Engineered Heart Tissue: Analysis of Contractile Force. Stem Cell Rep..

[74] Schmittgen T.D., Livak K.J. (2008). Analyzing real-time PCR data by the comparative C(T) method. Nat. Protoc..

[75] Ye J., Coulouris G., Zaretskaya I., Cutcutache I., Rozen S., Madden T.L. (2012). Primer-BLAST: a tool to design target-specific primers for polymerase chain reaction. BMC Bioinf..

[76] Niu G., Yang Y., Zhang Y., Hua C., Wang Z., Tang Z., Li K. (2016). Identifying suitable reference genes for gene expression analysis in developing skeletal muscle in pigs. PeerJ.

[77] Shao X., Taha I.N., Clauser K.R., Gao Y.T., Naba A. (2020). MatrisomeDB: the ECM-protein knowledge database. Nucleic Acids Res..

[78] Huang da W., Sherman B.T., Lempicki R.A. (2009). Systematic and integrative analysis of large gene lists using DAVID bioinformatics resources. Nat. Protoc..

[79] Huang da W., Sherman B.T., Lempicki R.A. (2009). Bioinformatics enrichment tools: paths toward the comprehensive functional analysis of large gene lists. Nucleic Acids Res..

[80] Gustavsen J.A., Pai S., Isserlin R., Demchak B., Pico A.R. (2019). RCy3: Network biology using Cytoscape from within R. F1000Res..

[81] Doncheva N.T., Morris J.H., Gorodkin J., Jensen L.J. (2019). Cytoscape StringApp: Network Analysis and Visualization of Proteomics Data. J. Proteome Res..

