# Supplementary material for: Extracellular Matrix Profiling and Disease Modelling in Engineered Vascular Smooth Muscle Cell Tissues
Source: Matrix Biol Plus. 2022 Sep 17;16:100122. doi: 10.1016/j.mbplus.2022.100122 (PMC9526190; doi:10.1016/j.mbplus.2022.100122)
Supplement: Supplementary data 1 [file mmc1.pdf]

SUPPLEMENTARY MATERIAL

Extracellular Matrix Profiling and Disease Modelling in Engineered Vascular Smooth Muscle Cell Tissues

Ella Reed[1], Adam Fellows[1,2], Ruifang Lu[1], Marieke Rienks[1], Lukas Schmidt[1], Xiaoke Yin[1], Elisa Durogetti[1], Mona Brandt[3], Susanne Krasemann[3], Kristin Hartmann[3], Javier Barallobre-Barreiro[1], Owen Addison[4], Friederike Cuello[3], Arne Hansen[3], Manuel Mayr[1].

[1]King's British Heart Foundation Centre, School of Cardiovascular Medicine and Sciences, London, SE5 9NU, UK.

[2]National Heart and Lung Institute, Imperial College London, Du Cane Road, London, W12 0NN, UK.

[3]Institute of Experimental Pharmacology and Toxicology, University Medical Center Hamburg-Eppendorf, German Center for Heart Research (DZHK), Hamburg 20251, DE.

[4]Centre of Oral, Clinical & Translational Sciences, Faculty of Dentistry, Oral & Craniofacial Sciences, King's College London, Guy's Hospital, London SE1 9RT, UK.

Corresponding Author Manuel Mayr, MD, PhD
King's British Heart Foundation Centre, King's College London
125 Coldharbour Lane, London SE5 9NU
United Kingdom
Email: manuel.mayr@kcl.ac.uk

Supplementary Table 1. ECM proteomics results for EVTs cultured for 1, 7 and 14 days*.

					Log2 transformed protein abundances ± SD			p values	
Category	Description	Gene	Accession	Coverage [%]	Day 1	Day 7	Day 14	Day 7 / Day 1	Day 14 / Day 1
Collagens	Collagen alpha-1(I) chain	Col1a1	P11087	58	3.89±1	4.43±0.55	5.24±2.06	0.464	0.365
Collagens	Collagen alpha-1(II) chain	Col2a1	P28481	3	3.59±2.98	4.77±0.42	3.96±3.47	0.533	0.895
Collagens	Collagen alpha-1(III) chain	Col3a1	P08121	7	3.13±2.7	3.79±3.3	6.02±1.42	0.802	0.176
Collagens	Collagen alpha-1(IV) chain	Col4a1	P02463	6	4.09±1.96	6.05±1.42	8.46±0.23	0.234	**0.019**
Collagens	Collagen alpha-1(V) chain	Col5a1	O88207	7	3.84±0.39	5.23±5.78	3.72±3.2	0.698	0.952
Collagens	Collagen alpha-1(VI) chain	Col6a1	Q04857	14	6.21±1.52	7.16±0.39	7.43±0.88	0.353	0.294
Collagens	Collagen alpha-1(XIV) chain	Col14a1	Q80X19	4	5.28±4.44	5.21±4.39	5.4±4.55	0.985	0.974
Collagens	Collagen alpha-1(XV) chain	Col15a1	O35206	8	6.65±0.49	7.01±0.37	8.25±0.96	0.370	0.061
Collagens	Collagen alpha-2(I) chain	Col1a2	Q01149	50	3.08±0.75	4.22±1.31	5.53±1.89	0.259	0.105
Collagens	Collagen alpha-2(IV) chain	Col4a2	P08122	5	2.28±3.69	2.08±3.34	8.7±0.31	0.947	**0.040**
Collagens	Collagen alpha-2(V) chain	Col5a2	Q3U962	13	5.68±1.26	5.87±0.39	6.61±0.33	0.809	0.285
Collagens	Collagen alpha-2(VI) chain	Col6a2	Q02788	6	4.32±3.61	2.64±4.31	4.12±3.44	0.633	0.948
Collagens	Collagen alpha-3(IV) chain	Col4a3	Q9QZS0	1	4.81±0.56	6.04±0.62	8.42±0.05	0.063	**0.000**
ECM Glycoproteins	Adipocyte enhancer-binding protein 1	Aebp1	Q640N1	15	5.64±0.53	7.11±0.47	7.29±0.57	**0.023**	**0.022**
ECM Glycoproteins	EGF-containing fibulin-like extracellular matrix protein 1	Efemp1	Q8BPB5	31	6.32±1.34	6.81±1.64	7.24±1.98	0.712	0.543
ECM Glycoproteins	EGF-containing fibulin-like extracellular matrix protein 2	Efemp2	Q9WVJ9	22	4.8±0.9	7.25±0.21	8.27±1.11	**0.010**	**0.014**
ECM Glycoproteins	EMILIN-1	Emilin1	Q99K41	25	6.11±0.64	6.72±0.5	7.99±0.65	0.264	**0.023**
ECM Glycoproteins	Extracellular matrix protein 1	Ecm1	Q61508	9	5.31±1.29	5.42±1.55	6.26±0.9	0.931	0.357
ECM Glycoproteins	Fibrillin-1	Fbn1	Q61554	4	5.17±1.7	6.7±1.3	5.66±1.16	0.284	0.701
ECM Glycoproteins	Fibronectin	Fn1	P11276	30	5.97±0.58	6.86±0.45	7.27±0.56	0.106	0.050
ECM Glycoproteins	Fibulin-2	Fbln2	P37889	54	4.74±0.99	6.15±0.85	6.91±1.03	0.136	0.058
ECM Glycoproteins	Fibulin-5	Fbln5	Q9WVH9	37	3.58±0.63	5.64±1.46	7.47±1.72	0.088	**0.021**
ECM Glycoproteins	Growth arrest-specific protein 6	Gas6	Q61592	18	1.16±1.74	5.11±2.25	7.31±2.83	0.074	**0.033**
ECM Glycoproteins	Hemicentin-1	Hmcn1	D3YXG0	1	6.99±0.69	7.4±0.55	7.47±0.63	0.463	0.422
ECM Glycoproteins	Insulin-like growth factor-binding protein 7	Igfbp7	Q61581	40	6.76±0.27	6.25±0.51	6.08±0.37	0.199	0.063
ECM Glycoproteins	Lactadherin	Mfge8	P21956	46	5.33±0.14	6.88±0.39	8.13±1.04	**0.003**	**0.010**
ECM Glycoproteins	Laminin subunit alpha-4	Lama4	P97927	2	6.32±0.53	7.19±0.71	8.16±0.32	0.167	**0.007**

ECM Glycoproteins	Laminin subunit alpha-5	Lama5	Q61001	4	7.05±0.83	6.58±1.05	6.89±1.19	0.575	0.860
ECM Glycoproteins	Laminin subunit beta-1	Lamb1	P02469	17	6.72±0.41	7.31±0.34	7.73±0.5	0.126	0.054
ECM Glycoproteins	Laminin subunit beta-2	Lamb2	Q61292	9	6.15±1.88	5.7±1.23	6.41±1.25	0.749	0.854
ECM Glycoproteins	Laminin subunit gamma-1	Lamc1	P02468	7	6.31±1.04	6.93±0.67	7.71±0.64	0.434	0.119
ECM Glycoproteins	Latent-transforming growth factor beta-binding protein 1	Ltbp1	Q8CG19	7	4.82±1.41	6.77±0.06	7.37±0.78	0.074	0.051
ECM Glycoproteins	Latent-transforming growth factor beta-binding protein 3	Ltbp3	Q61810	4	7.16±0.53	6.15±0.77	6.62±0.92	0.135	0.428
ECM Glycoproteins	Latent-transforming growth factor beta-binding protein 4	Ltbp4	Q8K4G1	4	2.65±4.32	2.73±4.47	8.95±1.94	0.982	0.083
ECM Glycoproteins	Matrix Gla protein	Mgp	P19788	32	3.08±2.63	6.4±0.45	5.95±0.58	0.098	0.139
ECM Glycoproteins	Nidogen-1	Nid1	P10493	26	6.92±0.26	7.81±0.67	8.36±0.56	0.096	**0.016**
ECM Glycoproteins	Nidogen-2	Nid2	O88322	4	6.52±0.21	6.96±1.24	2.17±3.5	0.578	0.098
ECM Glycoproteins	Osteopontin	Spp1	P10923	4	7.55±1.02	5.62±5.17	2.15±3.46	0.561	0.060
ECM Glycoproteins	Procollagen C-endopeptidase enhancer 1	Pcolce	Q61398	18	3.91±3.39	3.27±2.75	1.78±2.82	0.811	0.450
ECM Glycoproteins	Protein disulfide isomerase Creld1	Creld1	Q91XD7	13	6.34±1.33	7.12±0.93	7.61±1.65	0.455	0.357
ECM Glycoproteins	Protein disulfide isomerase Creld2	Creld2	Q9CYA0	12	6.73±0.52	6.67±0.2	7.04±0.57	0.868	0.522
ECM Glycoproteins	Slit homolog 3 protein	Slit3	Q9WVB4	16	6.12±0.31	4.61±0.88	2.92±2.52	**0.048**	0.094
ECM Glycoproteins	SPARC	Sparc	P07214	24	4.77±0.49	4.78±0.38	5.58±1.54	0.966	0.430
ECM Glycoproteins	SPARC-related modular calcium-binding protein 1	Smoc1	Q8BLY1	29	1.93±1.6	5.16±0.44	6.35±0.16	**0.028**	**0.009**
ECM Glycoproteins	Tenascin	Tnc	Q80YX1	17	4.74±0.43	5.31±1.18	4.27±4.15	0.480	0.855
ECM Glycoproteins	Thrombospondin-1	Thbs1	P35441	29	6.15±1.25	5.8±0.45	6.53±0.45	0.670	0.653
ECM Glycoproteins	von Willebrand factor A domain-containing protein 5A	Vwa5a	Q99KC8	34	6.58±0.21	6.95±0.23	7.09±0.27	0.111	0.061
ECM Regulators	72 kDa type IV collagenase	Mmp2	P33434	12	0.15±0	0.15±0	5.97±5.05	N/A	0.117
ECM Regulators	Alpha-1-antitrypsin 1-1	Serpina1a	P07758	55	5.74±1.53	7.18±2.43	5.16±0.4	0.433	0.564
ECM Regulators	Alpha-1-antitrypsin 1-2	Serpina1b	P22599	62	4.72±3.24	6.3±4.19	2.57±0.64	0.632	0.324
ECM Regulators	Alpha-1-antitrypsin 1-4	Serpina1d	Q00897	53	5.73±2.4	6.44±3.33	4.59±0.14	0.779	0.456
ECM Regulators	Alpha-1-antitrypsin 1-5	Serpina1e	Q00898	42	5.47±2.57	7.25±2.74	2.08±2.04	0.457	0.147
ECM Regulators	Antithrombin-III	Serpinc1	P32261	37	6.11±1.69	7.02±1.93	3.72±3.85	0.573	0.380
ECM Regulators	Bone morphogenetic protein 1	Bmp1	P98063	21	5.27±1.29	7.6±1.12	8.39±1.55	0.077	0.055
ECM Regulators	Cathepsin B	Ctsb	P10605	45	6.42±0.34	7.18±0.55	7.52±0.68	0.113	0.067
ECM Regulators	Cathepsin D	Ctsd	P18242	48	6.56±0.39	7.02±0.37	7.4±0.31	0.205	**0.042**
ECM Regulators	Cathepsin L1	Ctsl	P06797	35	4.6±1.42	6.22±1.17	7.45±0.89	0.201	**0.042**
ECM Regulators	Cathepsin Z	Ctsz	Q9WUU7	34	6.07±0.04	6.9±0.45	7.56±0.24	**0.033**	**0.000**

ECM Regulators	CD109 antigen	Cd109	Q8R422	24	7.23±0.71	6.55±0.95	6.73±1.41	0.378	0.617
ECM Regulators	Cystatin-B	Cstb	Q62426	70	6.64±0.44	6.53±0.32	6.76±0.32	0.753	0.721
ECM Regulators	Cystatin-C	Cst3	P21460	42	4.79±0.51	6.32±0.53	7.06±0.31	**0.023**	**0.003**
ECM Regulators	Disintegrin and metalloproteinase domain-containing protein 10	Adam10	O35598	12	7.21±0.63	6.53±0.51	6.56±0.73	0.217	0.304
ECM Regulators	Disintegrin and metalloproteinase domain-containing protein 17	Adam17	Q9Z0F8	2	6.39±0.1	5.8±0.52	5.58±0.27	0.123	**0.008**
ECM Regulators	Disintegrin and metalloproteinase domain-containing protein 9	Adam9	Q61072	3	7.64±0.82	4.15±3.47	0.15±0	0.166	**0.000**
ECM Regulators	Egl nine homolog 1	Egln1	Q91YE3	16	6.45±1.49	6.3±1.37	6.04±1.01	0.903	0.713
ECM Regulators	Histidine-rich glycoprotein	Hrg	Q9ESB3	10	3.23±5.34	8.41±1.27	5.48±4.62	0.178	0.610
ECM Regulators	Inter-alpha-trypsin inhibitor heavy chain H2	Itih2	Q61703	18	5.88±0.66	6.71±0.53	7.43±0.36	0.166	**0.023**
ECM Regulators	Kininogen-1	Kng1	O08677	19	6.55±1.46	7.9±1.73	6.52±0.52	0.361	0.973
ECM Regulators	Leukocyte elastase inhibitor A	Serpinb1a	Q9D154	60	6.03±1.76	6.28±1.71	6.27±1.69	0.868	0.874
ECM Regulators	Lysosomal protective protein	Ctsa	P16675	27	6.32±0.33	7.14±0.26	7.67±0.54	**0.029**	**0.021**
ECM Regulators	Lysyl oxidase homolog 2	Loxl2	P58022	4	6.54±5.53	3.33±5.51	2.63±4.29	0.517	0.389
ECM Regulators	Matrix metalloproteinase-14	Mmp14	P53690	6	7.26±0.66	4.42±3.7	4.76±4	0.260	0.345
ECM Regulators	Metalloproteinase inhibitor 2	Timp2	P25785	17	4.8±0.99	5.96±1.15	6.76±0.93	0.257	0.067
ECM Regulators	Multifunctional procollagen lysine hydroxylase and glycosyltransferase LH3	Plod3	Q9R0E1	21	6.54±0.5	6.28±0.05	5.54±1.12	0.419	0.233
ECM Regulators	Peptide-N(4)-(N-acetyl-beta-glucosaminyl)asparagine amidase	Ngly1	Q9JI78	5	6.53±0.14	7.13±0.58	7.49±0.21	0.160	**0.003**
ECM Regulators	Plasminogen	Plg	P20918	13	4.85±2.45	5.75±3.11	5.85±1.39	0.714	0.570
ECM Regulators	Plasminogen activator inhibitor 1	Serpine1	P22777	5	6.81±1.6	2.51±4.09	0.15±0	0.165	**0.002**
ECM Regulators	Pregnancy zone protein	Pzp	Q61838	28	6.12±0.61	6.94±1.68	6.4±0.36	0.472	0.530
ECM Regulators	Pro-cathepsin H	Ctsh	P49935	41	5.98±0.35	7.1±0.26	8.01±0.22	**0.011**	**0.001**
ECM Regulators	Prolyl 3-hydroxylase OGFOD1	Ogfod1	Q3U0K8	3	6.5±1.19	7.54±1.17	8.04±0.79	0.342	0.136
ECM Regulators	Prolyl 4-hydroxylase subunit alpha-1	P4ha1	Q60715	63	6.88±0.58	6.68±0.07	6.62±0.11	0.583	0.480
ECM Regulators	Prolyl 4-hydroxylase subunit alpha-2	P4ha2	Q60716	49	7.52±0.79	7.26±0.56	6.39±0.58	0.662	0.114
ECM Regulators	Protein-glutamine gamma-glutamyltransferase 2	Tgm2	P21981	52	5.29±1.69	5.63±0.98	6.48±1.93	0.776	0.467
ECM Regulators	Protein-lysine 6-oxidase	Lox	P28301	35	3.35±3.32	3.66±3.14	4.66±1.88	0.911	0.584
ECM Regulators	Serine protease HTRA1	Htra1	Q9R118	36	4.99±1.11	5.59±1.13	6.97±0.85	0.545	0.070
ECM Regulators	Serine protease inhibitor A3K	Serpina3k	P07759	32	5.36±2.13	6.58±3.46	4.6±0.79	0.629	0.592
ECM Regulators	Serine protease inhibitor A3N	Serpina3n	Q91WP6	20	4.75±3.99	5.09±4.31	2.73±4.47	0.926	0.590
ECM Regulators	Serpin B8	Serpinb8	O08800	17	6.13±0.8	6.17±0.49	7.18±0.8	0.952	0.184

ECM Regulators	Serpin H1	Serpinh1	P19324	60	6.48±0.56	5.9±0.59	5.44±0.57	0.280	0.086
ECM Regulators	Stromelysin-1	Mmp3	P28862	20	6.46±1.59	7.39±2.16	5.96±0.51	0.580	0.628
ECM-affiliated Proteins	Annexin A1	Anxa1	P10107	86	6.82±0.14	6.51±0.36	6.56±0.37	0.237	0.328
ECM-affiliated Proteins	Annexin A11	Anxa11	P97384	41	6.78±0.57	6.78±0.53	7.22±0.6	0.992	0.407
ECM-affiliated Proteins	Annexin A2	Anxa2	P07356	84	6.76±0.34	6.66±0.26	6.44±0.37	0.694	0.324
ECM-affiliated Proteins	Annexin A3	Anxa3	O35639	84	7.16±0.07	6.31±0.09	5.89±0.17	**0.000**	**0.000**
ECM-affiliated Proteins	Annexin A4	Anxa4	P97429	79	6.69±0.44	6.82±0.33	7.22±0.69	0.708	0.325
ECM-affiliated Proteins	Annexin A5	Anxa5	P48036	86	6.95±0.17	6.63±0.29	6.99±0.37	0.181	0.859
ECM-affiliated Proteins	Annexin A6	Anxa6	P14824	82	6.78±0.06	6.38±0.3	6.83±0.55	0.089	0.875
ECM-affiliated Proteins	Annexin A7	Anxa7	Q07076	49	6.69±0.38	6.93±0.35	7.33±0.3	0.453	0.084
ECM-affiliated Proteins	Annexin A8	Anxa8	O35640	71	7.56±0.55	7.09±0.12	6.28±0.42	0.230	**0.034**
ECM-affiliated Proteins	Galectin-1	Lgals1	P16045	75	6.86±0.36	6.31±0.3	6.64±0.6	0.107	0.617
ECM-affiliated Proteins	Galectin-3	Lgals3	P16110	39	7.21±0.15	7.31±0.14	6.7±0.21	0.448	**0.026**
ECM-affiliated Proteins	Galectin-9	Lgals9	O08573	7	2.08±3.34	1.96±3.14	4.21±3.53	0.968	0.490
ECM-affiliated Proteins	Galectin-related protein	Lgalsl	Q8VED9	6	7.6±0.42	5.54±4.67	8.41±0.9	0.489	0.233
ECM-affiliated Proteins	Glypican-1	Gpc1	Q9QZF2	18	7.23±1.75	6.38±1.31	5.94±1.64	0.538	0.404
ECM-affiliated Proteins	Glypican-4	Gpc4	P51655	11	5.27±0.89	7.15±0.31	7.54±0.48	**0.026**	**0.018**
ECM-affiliated Proteins	Glypican-6	Gpc6	Q9R087	33	4.88±1.76	5.91±0.3	6.74±0.76	0.376	0.170
ECM-affiliated Proteins	Hemopexin	Hpx	Q91X72	35	6.15±1.99	7.16±3.02	3.23±2.75	0.651	0.211
ECM-affiliated Proteins	Plexin domain-containing protein 2	Plxdc2	Q9DC11	26	4.95±0.86	5.33±0.78	4.49±0.87	0.601	0.553
ECM-affiliated Proteins	Plexin-B2	Plxnb2	B2RXS4	25	6.56±0.13	6.53±0.13	6.66±0.38	0.790	0.688
ECM-affiliated Proteins	Protein ERGIC-53	Lman1	Q9D0F3	36	6.97±0.37	7.09±0.34	7.05±0.51	0.706	0.830
ECM-affiliated Proteins	Syndecan-1	Sdc1	P18828	10	4.47±3.93	4.55±3.81	4.55±3.82	0.980	0.980
ECM-affiliated Proteins	Syndecan-2	Sdc2	P43407	5	5.75±0.36	5.77±0.85	6.19±0.54	0.965	0.303
ECM-affiliated Proteins	Syndecan-4	Sdc4	O35988	15	7.6±0.73	7.29±1.85	4.34±3.64	0.801	0.203
Proteoglycans	Basement membrane-specific heparan sulfate proteoglycan core protein	Hspg2	Q05793	17	7.65±1.35	7.56±1.59	8.41±0.44	0.947	0.406
Proteoglycans	Biglycan	Bgn	P28653	40	4.47±0.81	6.41±0.49	7.15±0.58	**0.024**	**0.010**
Proteoglycans	Chondroitin sulfate proteoglycan 4	Cspg4	Q8VHY0	37	6.58±0.55	6.61±0.31	6.46±0.33	0.927	0.769
Proteoglycans	Mimecan	Ogn	Q62000	17	0.15±0	0.15±0	7.19±2.38	N/A	**0.007**
Proteoglycans	Prolargin	Prelp	Q9JK53	7	0.15±0	7.04±0.88	8.72±0.66	**0.000**	**0.000**
Proteoglycans	Proteoglycan 4	Prg4	Q9JM99	6	4.96±1	7.14±2.13	6.69±0.37	0.184	**0.048**
Proteoglycans	Versican core protein	Vcan	Q62059	11	4.58±0.44	8.21±0.56	8.94±0.98	**0.001**	**0.002**

Secreted Factors	Angiopoietin-2	Angpt2	O35608	10	3.9±3.26	3.34±2.79	4.97±4.35	0.833	0.751
Secreted Factors	C-X-C motif chemokine 15	Cxcl15	Q9WVL7	10	2.17±3.49	4.51±3.81	2.21±3.56	0.476	0.989
Secreted Factors	Cytokine receptor-like factor 3	Crlf3	Q9Z2L7	10	7.72±0.62	5.88±0.92	5.51±0.29	**0.046**	**0.005**
Secreted Factors	Follistatin-related protein 1	Fstl1	Q62356	28	6.17±0.59	6.54±0.4	7.11±0.77	0.424	0.169
Secreted Factors	Fractalkine	Cx3cl1	O35188	33	6.04±1.27	6.97±1.08	6.67±2.03	0.388	0.670
Secreted Factors	Hepatocyte growth factor activator	Hgfac	Q9R098	2	2.38±3.87	2.73±4.46	2.72±4.45	0.924	0.926
Secreted Factors	Host cell factor 1	Hcfc1	Q61191	7	6.54±0.09	6.83±0.15	6.87±0.44	0.052	0.284
Secreted Factors	Macrophage colony-stimulating factor 1	Csf1	P07141	9	4.03±3.37	6.54±0.4	7.34±0.98	0.270	0.178
Secreted Factors	Protein S100-A1	S100a1	P56565	23	4.57±3.84	7.35±0.24	8.02±0.55	0.279	0.199
Secreted Factors	Protein S100-A10	S100a10	P08207	62	6.89±0.15	6.5±0.27	6.81±0.37	0.093	0.750
Secreted Factors	Protein S100-A11	S100a11	P50543	57	6.78±0.08	6.29±0.32	6.3±0.45	0.064	0.145
Secreted Factors	Protein S100-A13	S100a13	P97352	48	6.45±0.16	6.57±0.09	7.05±0.62	0.310	0.177
Secreted Factors	Protein S100-A16	S100a16	Q9D708	10	6.52±0.11	6.42±0.41	6.96±0.23	0.698	**0.039**
Secreted Factors	Protein S100-A4	S100a4	P07091	64	6.57±1.26	5.9±1.28	6.1±1.71	0.556	0.726
Secreted Factors	Protein S100-A6	S100a6	P14069	52	6.78±0.07	6.48±0.19	6.4±0.16	0.065	**0.021**

*ECM proteins were selected utilising the Matrisome DB annotator tool and manual selection. N=3 biological replicates and N=2 technical replicates per condition. Significance compared to control group was determined by unpaired Student's t-test with a p value <0.05 considered significant. SD = standard deviation. Missing values were imputed.

Supplementary Table 2. ECM proteomics results for control EVTs and EVTs treated with TGFβ-1 (2 or 10 ng / mL) and ALKi (10 µM) for 7 days*.

					Log2 transformed protein abundances ± SD				p values		
Category	Description	Gene	Accession	Coverage [%]	Control	TGFβ-1 (2 ng / mL)	TGFβ-1 (10 ng / mL)	ALKI (10 µM)	TGFβ-1 (2 ng / mL) / Control	TGFβ-1 (10 ng / mL) / Control	ALKi (10 µM) / Control
Collagens	Collagen alpha-1(I) chain	Col1a1	P11087	68	6.59±0.68	7.03±0.4	7.28±0.35	5.51±0.58	0.390	0.194	0.104
Collagens	Collagen alpha-1(III) chain	Col3a1	P08121	20	5.71±1.07	6.39±2.08	6.93±1.97	4.9±0.78	0.643	0.399	0.344
Collagens	Collagen alpha-1(IV) chain	Col4a1	P02463	8	7.18±0.35	6.52±0.81	6.34±0.81	6.39±0.72	0.259	0.173	0.163
Collagens	Collagen alpha-1(V) chain	Col5a1	O88207	3	6.67±0.92	6.84±1.22	6.64±0.68	6.17±0.25	0.857	0.969	0.418
Collagens	Collagen alpha-1(VI) chain	Col6a1	Q04857	11	6.94±0.52	6.69±1.16	6.42±0.85	5.59±0.42	0.747	0.421	**0.025**
Collagens	Collagen alpha-1(VIII) chain	Col8a1	Q00780	3	6.71±1.68	5.73±1.71	5.78±1.79	6.74±0.86	0.517	0.549	0.978
Collagens	Collagen alpha-1(XIV) chain	Col14a1	Q80X19	42	5.74±1.38	6.37±2.18	6.23±1.85	5.65±1.51	0.695	0.731	0.943
Collagens	Collagen alpha-1(XV) chain	Col15a1	O35206	4	6.49±0.86	5.92±1.5	6.09±1.36	5.65±0.66	0.598	0.687	0.250
Collagens	Collagen alpha-1(XVIII) chain	Col18a1	P39061	4	1.86±2.96	6.3±0.89	6.9±1.97	2.12±3.41	0.068	0.070	0.927
Collagens	Collagen alpha-2(I) chain	Col1a2	Q01149	60	6.25±0.83	6.93±0.62	7.22±0.39	5.57±0.54	0.324	0.144	0.297
Collagens	Collagen alpha-2(IV) chain	Col4a2	P08122	7	6.92±0.43	6.74±0.5	6.92±0.52	6.35±0.41	0.665	0.991	0.176
Collagens	Collagen alpha-2(V) chain	Col5a2	Q3U962	8	4.79±0.2	6.96±0.11	8.28±0.52	4.19±0.41	**0.000**	**0.000**	0.087
Collagens	Collagen alpha-3(IV) chain	Col4a3	Q9QZS0	3	7.25±0.12	6.44±0.3	6.25±0.37	6.78±0.35	**0.013**	**0.011**	0.092
ECM Glycoproteins	Adipocyte enhancer-binding protein 1	Aebp1	Q640N1	16	4.57±1.36	7.2±0.72	7.48±0.11	5.22±1.83	**0.042**	**0.021**	0.648
ECM Glycoproteins	Agrin	Agrn	A2ASQ1	5	5.09±0.62	6.79±1.41	7.8±0.78	4.5±0.96	0.128	**0.009**	0.426
ECM Glycoproteins	Cartilage oligomeric matrix protein	Comp	Q9R0G6	3	7.24±1.02	5.6±0.66	5.97±0.32	7.13±0.58	0.080	0.108	0.880
ECM Glycoproteins	EGF-containing fibulin-like extracellular matrix protein 1	Efemp1	Q8BPB5	40	5.84±1.63	6.97±1.77	5.71±2.08	5.39±1.21	0.459	0.936	0.725
ECM Glycoproteins	EGF-containing fibulin-like extracellular matrix protein 2	Efemp2	Q9WVJ9	32	5.42±0.66	7.46±0.33	7.4±0.54	4.57±0.51	**0.009**	**0.016**	0.150
ECM Glycoproteins	Elastin	Eln	P54320	13	1.59±2.48	6.3±1.16	8.09±1.51	0.15±0	0.094	**0.020**	0.374
ECM Glycoproteins	EMILIN-1	Emilin1	Q99K41	25	5.66±0.57	7.47±0.72	7.28±0.29	4.19±0.75	**0.027**	**0.012**	**0.054**
ECM Glycoproteins	Extracellular matrix protein 1	Ecm1	Q61508	7	6.86±0.3	6.72±0.26	6.81±0.31	6.4±0.37	0.573	0.834	0.168
ECM Glycoproteins	Fibrillin-1	Fbn1	Q61554	5	6.46±0.38	6.89±0.35	7.4±0.4	5.34±0.49	0.231	**0.043**	**0.036**
ECM Glycoproteins	Fibrinogen gamma chain	Fgg	Q8VCM7	7	6.58±1.84	3.91±2.28	3.36±0.9	7.06±1.82	0.190	**0.053**	0.763
ECM Glycoproteins	Fibronectin	Fn1	P11276	57	5.15±0.33	7.01±1.36	6.79±1.52	4.27±0.22	0.083	0.141	**0.019**

ECM Glycoproteins	Fibulin-1	Fbln1	Q08879	13	5.03±0.49	7.68±1.16	6.8±0.78	3.12±2.69	**0.022**	**0.029**	0.294
ECM Glycoproteins	Fibulin-2	Fbln2	P37889	64	5.93±0.33	7.44±0.43	7.4±0.11	4.54±0.57	**0.009**	**0.002**	**0.022**
ECM Glycoproteins	Fibulin-5	Fbln5	Q9WVH9	28	5.65±1.49	7.54±1.01	7.59±0.47	2.76±1.37	0.144	0.098	0.069
ECM Glycoproteins	Growth arrest-specific protein 6	Gas6	Q61592	18	6.84±2.14	4.83±3.19	2.17±2.67	7.09±1.22	0.417	0.078	0.867
ECM Glycoproteins	Insulin-like growth factor-binding protein 7	Igfbp7	Q61581	48	5.72±0.94	6.76±0.61	7.38±0.65	5.24±0.74	0.182	0.066	0.526
ECM Glycoproteins	Lactadherin	Mfge8	P21956	54	6.77±0.47	6.26±0.46	6.94±0.57	6.63±0.7	0.253	0.713	0.796
ECM Glycoproteins	Laminin subunit alpha-5	Lama5	Q61001	7	5.37±0.53	6.79±0.75	7.44±0.31	5.01±0.31	**0.056**	**0.004**	0.371
ECM Glycoproteins	Laminin subunit beta-1	Lamb1	P02469	26	5.98±0.8	6.87±0.32	7.15±0.13	5.08±1.1	0.147	0.067	0.317
ECM Glycoproteins	Laminin subunit beta-2	Lamb2	Q61292	6	7.18±0.09	5.99±0.76	6.31±0.2	7.03±0.47	**0.055**	**0.002**	0.622
ECM Glycoproteins	Laminin subunit gamma-1	Lamc1	P02468	11	5.51±1.3	7.21±0.23	7.14±0.16	5.45±0.19	0.089	0.097	0.945
ECM Glycoproteins	Latent-transforming growth factor beta-binding protein 1	Ltbp1	Q8CG19	14	5.84±0.06	7.52±0.21	7.28±0.28	4.51±0.77	**0.000**	**0.001**	**0.041**
ECM Glycoproteins	Latent-transforming growth factor beta-binding protein 2	Ltbp2	O08999	9	1.21±1.83	7.58±0.29	8.15±0.74	0.15±0	**0.041**	**0.018**	0.374
ECM Glycoproteins	Latent-transforming growth factor beta-binding protein 3	Ltbp3	Q61810	9	6.42±0.09	6.95±0.43	7.07±0.13	5.38±0.16	0.105	**0.002**	**0.001**
ECM Glycoproteins	Latent-transforming growth factor beta-binding protein 4	Ltbp4	Q8K4G1	12	6.17±0.98	6.22±2.13	6.21±2.34	5.66±0.43	0.973	0.981	0.453
ECM Glycoproteins	Matrix Gla protein	Mgp	P19788	42	6.21±0.65	6.75±0.41	6.86±0.59	6.05±0.6	0.288	0.267	0.776
ECM Glycoproteins	Nidogen-1	Nid1	P10493	16	5.52±1.94	6.44±1.23	6.7±0.82	4.4±1.32	0.526	0.387	0.455
ECM Glycoproteins	Osteopontin	Spp1	P10923	21	2.95±1.69	6.13±1.05	7.67±1.34	3.08±2.64	**0.050**	**0.019**	0.946
ECM Glycoproteins	Procollagen C-endopeptidase enhancer 1	Pcolce	Q61398	24	6.61±1.02	6.92±0.96	7.11±0.25	3.44±2.92	0.719	0.457	0.151
ECM Glycoproteins	Protein disulfide isomerase Creld1	Creld1	Q91XD7	19	6.5±0.62	6.65±0.34	6.65±0.65	6.67±0.41	0.722	0.785	0.715
ECM Glycoproteins	Protein disulfide isomerase Creld2	Creld2	Q9CYA0	15	6.45±0.36	6.75±0.14	6.86±0.4	6.38±0.37	0.249	0.266	0.832
ECM Glycoproteins	SPARC	Sparc	P07214	35	5.22±1.04	7.14±0.37	7.8±0.6	4.09±0.77	**0.039**	**0.020**	0.203
ECM Glycoproteins	SPARC-related modular calcium-binding protein 1	Smoc1	Q8BLY1	22	4.74±0.77	6.99±1.32	7.74±0.79	1.09±1.62	0.064	**0.009**	**0.025**
ECM Glycoproteins	Tenascin	Tnc	Q80YX1	4	1.79±2.84	6.36±2.24	8.08±0.59	0.15±0	**0.004**	**0.004**	0.374
ECM Glycoproteins	Thrombospondin-1	Thbs1	P35441	47	4.97±0.64	6.92±0.87	8.13±0.38	4.33±0.34	**0.035**	**0.002**	0.198
ECM Glycoproteins	Thrombospondin-2	Thbs2	Q03350	20	3.63±1.5	5.62±1.93	7.17±3.38	2.24±1.9	0.230	0.173	0.375
ECM Glycoproteins	von Willebrand factor A domain-containing protein 5A	Vwa5a	Q99KC8	47	6.63±0.52	6.78±0.22	6.58±0.23	6.6±0.48	0.664	0.892	0.953
ECM Regulators	Bone morphogenetic protein 1	Bmp1	P98063	29	6.26±1.08	7.23±0.53	7.19±0.56	5.33±1.29	0.234	0.259	0.394
ECM Regulators	Cathepsin B	Ctsb	P10605	56	6.81±0.53	6.52±0.43	6.61±0.52	6.67±0.56	0.508	0.671	0.770
ECM Regulators	Cathepsin D	Ctsd	P18242	73	6.65±0.42	6.58±0.35	6.62±0.62	6.86±0.34	0.848	0.959	0.527

ECM Regulators	Cathepsin Z	Ctsz	Q9WUU7	46	6.96±0.2	6.37±0.42	6.42±0.51	6.97±0.44	0.094	0.165	0.971
ECM Regulators	CD109 antigen	Cd109	Q8R422	7	7.12±1.45	4.66±2.65	4.27±3.87	7±0.83	0.232	0.298	0.909
ECM Regulators	Cystatin-B	Cstb	Q62426	70	6.41±0.58	6.55±0.63	6.75±0.77	6.63±0.4	0.787	0.576	0.614
ECM Regulators	Cystatin-C	Cst3	P21460	33	7.09±0.28	6.42±0.45	6.09±0.24	6.79±0.62	0.092	**0.009**	0.485
ECM Regulators	Disintegrin and metalloproteinase domain-containing protein 10	Adam10	O35598	22	7.26±0.55	5.83±0.77	5.29±0.54	7.26±0.48	**0.059**	**0.011**	0.992
ECM Regulators	Disintegrin and metalloproteinase domain-containing protein 17	Adam17	Q9Z0F8	7	6.77±0.03	6.3±0.2	6.18±0.51	6.84±0.37	**0.015**	0.111	0.764
ECM Regulators	Disintegrin and metalloproteinase domain-containing protein 9	Adam9	Q61072	8	6.97±0.35	6.32±0.44	5.56±0.25	6.85±0.65	0.116	**0.005**	0.802
ECM Regulators	Egl nine homolog 1	Egln1	Q91YE3	11	5.5±0.76	7.19±0.46	7.32±0.16	5.71±0.32	**0.031**	**0.016**	0.692
ECM Regulators	Extracellular sulfatase Sulf-1	Sulf1	Q8K007	3	0.15±0	7.18±0.99	7.91±1.34	0.15±0	**0.000**	**0.001**	N/A
ECM Regulators	Glia-derived nexin	Serpine2	Q07235	22	7.34±0.59	5.95±0.54	5.82±0.61	7.09±0.15	**0.039**	**0.035**	0.504
ECM Regulators	Inter-alpha-trypsin inhibitor heavy chain H2	Itih2	Q61703	17	6.89±0.47	6.19±0.05	5.64±0.71	7.16±0.46	0.063	0.064	0.515
ECM Regulators	Leukocyte elastase inhibitor A	Serpinb1a	Q9D154	58	6.18±1.18	6.65±1.27	6.76±1.25	5.9±1.49	0.662	0.586	0.812
ECM Regulators	Lysosomal protective protein	Ctsa	P16675	35	7.01±0.53	6.25±0.24	6.13±0.25	7.17±0.15	0.085	0.060	0.636
ECM Regulators	Lysyl oxidase homolog 2	Loxl2	P58022	14	3.8±2.05	6.26±1.71	8.19±0.47	5.08±2.7	0.187	**0.023**	0.551
ECM Regulators	Matrix metalloproteinase-14	Mmp14	P53690	8	4.88±1.02	6.41±0.99	7.66±1.33	5.1±0.68	0.134	**0.045**	0.763
ECM Regulators	Metalloproteinase inhibitor 1	Timp1	P12032	12	2.05±3.29	7.54±0.58	7.84±0.83	3.66±3.05	**0.047**	**0.042**	0.570
ECM Regulators	Multifunctional procollagen lysine hydroxylase and glycosyltransferase LH3	Plod3	Q9R0E1	27	6.19±0.52	6.66±0.87	6.86±1.09	5.83±0.43	0.461	0.386	0.414
ECM Regulators	Peptide-N(4)-(N-acetyl-beta-glucosaminyl)asparagine amidase	Ngly1	Q9JI78	6	7.22±0.37	4.55±3.81	6.5±0.18	7.22±0.16	0.295	**0.041**	0.977
ECM Regulators	Pro-cathepsin H	Ctsh	P49935	57	7.15±0.3	6.48±0.27	6.36±0.36	6.45±0.27	**0.046**	**0.043**	**0.041**
ECM Regulators	Procathepsin L	Ctsl	P06797	57	6.76±0.65	6.48±0.75	6.56±0.57	6.74±0.88	0.660	0.710	0.977
ECM Regulators	Prolyl 4-hydroxylase subunit alpha-1	P4ha1	Q60715	74	6.25±0.39	6.82±0.36	7.11±0.38	5.64±0.14	0.134	**0.052**	0.063
ECM Regulators	Prolyl 4-hydroxylase subunit alpha-2	P4ha2	Q60716	56	6.97±0.27	6.31±0.65	6.58±0.48	6.72±0.06	0.183	0.289	0.199
ECM Regulators	Protein-glutamine gamma-glutamyltransferase 2	Tgm2	P21981	38	6.4±1.23	6.44±1.67	5.83±1.08	5.87±1.3	0.972	0.581	0.637
ECM Regulators	Protein-lysine 6-oxidase	Lox	P28301	13	3.27±2.96	6.88±1.33	7.78±1.53	3.7±0.62	0.127	0.079	0.818
ECM Regulators	Serine protease HTRA1	Htra1	Q9R118	35	6.36±0.94	6.5±1.7	6.82±1.33	5.39±0.71	0.904	0.652	0.226
ECM Regulators	Serpin B8	Serpinb8	O08800	14	6.85±0.22	6.17±0.66	4.77±1.58	7.34±1.32	0.165	0.086	0.558

ECM Regulators	Serpin H1	Serpinh1	P19324	66	6.21±0.66	6.74±0.94	6.88±1.09	5.86±0.53	0.471	0.412	0.512
ECM Regulators	Stromelysin-1	Mmp3	P28862	24	5.43±0.84	7.03±1.28	7.09±1.28	5.35±0.48	0.143	0.134	0.890
ECM Regulators	Tolloid-like protein 1	Tll1	Q62381	5	6.35±0.36	6.98±0.83	6.62±0.15	6.64±0.21	0.298	0.311	0.305
ECM-affiliated Proteins	Annexin A1	Anxa1	P10107	88	6.9±0.38	6.44±0.55	6.52±0.41	6.75±0.35	0.299	0.307	0.652
ECM-affiliated Proteins	Annexin A11	Anxa11	P97384	40	7.06±0.66	6.26±0.89	5.89±0.87	6.72±0.77	0.279	0.137	0.593
ECM-affiliated Proteins	Annexin A2	Anxa2	P07356	86	6.76±0.23	6.44±0.08	6.46±0.19	6.51±0.13	0.088	0.160	0.180
ECM-affiliated Proteins	Annexin A3	Anxa3	O35639	84	6.85±0.02	6.81±0.32	6.62±0.15	6.61±0.09	0.848	**0.059**	**0.013**
ECM-affiliated Proteins	Annexin A4	Anxa4	P97429	65	7.11±0.63	6.2±0.64	5.98±0.56	7.05±0.46	0.155	0.081	0.898
ECM-affiliated Proteins	Annexin A5	Anxa5	P48036	95	6.69±0.46	6.74±0.77	7.02±0.49	6.33±0.23	0.931	0.440	0.303
ECM-affiliated Proteins	Annexin A6	Anxa6	P14824	84	7.09±0.15	6.52±0.18	6.5±0.17	6.72±0.19	**0.013**	**0.010**	**0.056**
ECM-affiliated Proteins	Annexin A7	Anxa7	Q07076	50	6.95±0.39	6.5±0.81	6.2±0.75	6.64±0.44	0.441	0.200	0.424
ECM-affiliated Proteins	Annexin A8	Anxa8	O35640	76	6.22±0.18	7.2±0.06	7.19±0.38	5.98±0.49	**0.001**	**0.016**	0.472
ECM-affiliated Proteins	C-type lectin domain family 2 member D	Clec2d	Q91V08	18	6.76±1.42	5.55±0.91	4.24±3.54	7.86±0.68	0.280	0.315	0.294
ECM-affiliated Proteins	Galectin-1	Lgals1	P16045	96	6.56±0.55	6.6±0.57	6.76±0.38	6.43±0.49	0.927	0.618	0.784
ECM-affiliated Proteins	Galectin-3	Lgals3	P16110	39	6.79±0.57	6.18±0.36	5.98±0.65	6.92±0.51	0.190	0.181	0.775
ECM-affiliated Proteins	Galectin-9	Lgals9	O08573	6	6.84±0.95	4.44±3.72	5.89±0.43	7.18±0.66	0.341	0.192	0.635
ECM-affiliated Proteins	Glypican-1	Gpc1	Q9QZF2	27	5.95±0.66	6.67±0.24	7.11±0.22	6.29±0.73	0.153	**0.045**	0.590
ECM-affiliated Proteins	Glypican-4	Gpc4	P51655	34	6.67±0.72	6.77±0.52	6.13±0.67	6.51±0.5	0.850	0.401	0.773
ECM-affiliated Proteins	Glypican-6	Gpc6	Q9R087	28	7.18±0.78	6.34±0.49	5.2±0.53	7.29±0.71	0.191	**0.022**	0.857
ECM-affiliated Proteins	Gremlin-1	Grem1	O70326	51	3.55±2.94	6.13±2.07	4.43±1.56	3.56±2.97	0.282	0.668	0.996
ECM-affiliated Proteins	Plexin-B2	Plxnb2	B2RXS4	26	6.96±0.06	6.4±0.23	6.25±0.2	6.8±0.15	**0.015**	**0.004**	0.160
ECM-affiliated Proteins	Protein ERGIC-53	Lman1	Q9D0F3	54	6.56±0.28	6.63±0.29	6.77±0.5	6.26±0.24	0.769	0.558	0.229
ECM-affiliated Proteins	Syndecan-4	Sdc4	O35988	15	4.65±3.89	7.27±0.81	6.97±0.76	3.89±3.26	0.316	0.369	0.809
Proteoglycans	Basement membrane-specific heparan sulfate proteoglycan core protein	Hspg2	Q05793	31	5.37±0.34	7.34±0.4	7.21±0.84	4.8±0.61	**0.003**	**0.024**	0.231
Proteoglycans	Biglycan	Bgn	P28653	59	5.46±0.86	6.99±0.38	7.66±0.66	4.13±0.92	**0.049**	**0.025**	0.140
Proteoglycans	Chondroitin sulfate proteoglycan 4	Cspg4	Q8VHY0	46	5.93±0.43	6.73±0.7	7.6±0.23	5.28±0.3	0.167	0.004	0.097
Proteoglycans	Decorin	Dcn	P28654	21	6±1.17	6.95±0.68	6.97±0.88	5.82±0.94	0.295	0.317	0.845
Proteoglycans	Versican core protein	Vcan	Q62059	8	3.45±2.03	6.96±1.65	7.51±1.54	2.95±1.26	0.081	**0.051**	0.736
Secreted Factors	Angiopoietin-2	Angpt2	O35608	15	6.54±2.42	4.68±3.92	4.95±0.37	6.78±2.05	0.524	0.323	0.902
Secreted Factors	Follistatin-related protein 1	Fstl1	Q62356	17	5.66±1.39	6.52±1.13	7.81±0.61	3.3±2.86	0.451	0.070	0.269

Secreted Factors	Fractalkine	Cx3cl1	O35188	15	5.91±1.39	6.81±1	7.12±0.78	5.5±1.14	0.412	0.256	0.713
Secreted Factors	Host cell factor 1	Hcfc1	Q61191	17	6.69±0.36	6.54±0.26	6.48±0.5	6.58±0.51	0.583	0.586	0.776
Secreted Factors	Macrophage colony-stimulating factor 1	Csf1	P07141	5	6.27±1.49	6.31±0.9	6.44±0.59	6.62±0.98	0.976	0.864	0.753
Secreted Factors	Multiple epidermal growth factor-like domains protein 8	Megf8	P60882	1	6.18±0.38	6.7±0.92	6.83±0.7	6.53±0.51	0.413	0.232	0.391
Secreted Factors	Protein S100-A10	S100a10	P08207	62	7.2±0.2	6.33±0.28	6.14±0.32	6.56±0.32	**0.011**	**0.008**	**0.042**
Secreted Factors	Protein S100-A11	S100a11	P50543	95	6.84±0.19	6.73±0.19	6.55±0.15	6.74±0.22	0.504	0.096	0.584
Secreted Factors	Protein S100-A13	S100a13	P97352	49	6.86±0.75	6.29±0.73	6.32±0.51	6.45±0.39	0.392	0.357	0.442
Secreted Factors	Protein S100-A4	S100a4	P07091	64	6.81±0.99	6.33±0.99	6.25±1.15	6.47±0.86	0.580	0.556	0.677
Secreted Factors	Protein S100-A6	S100a6	P14069	80	6.74±0.27	6.56±0.62	6.45±0.47	6.79±0.47	0.667	0.398	0.897
Secreted Factors	Tumor necrosis factor receptor superfamily member 11B	Tnfrsf11b	O08712	19	0.15±0	2.98±4.9	7.33±2.28	0.15±0	0.374	**0.006**	N/A

* ECM proteins were selected utilising the Matrisome DB annotator tool and manual selection. N=3 biological replicates and N=2 technical replicates per condition. Significance compared to control group was determined by unpaired Student's t-test with a p value <0.05 considered significant. SD = standard deviation. Missing values were imputed.

Supplementary Table 3. Receptors identified in the proteomics analysis of control EVT lysates, and EVT lysates treated with TGFβ-1 (2 or 10 ng / mL) and ALKi (10 μM) for 7 days*.

					Log2 transformed protein abundances ± SD				p values		
Category	Description	Gene	Accession	Coverage [%]	Control	TGFβ-1 (2 ng / mL)	TGFβ-1 (10 ng / mL)	ALKi (10 μM)	TGFβ-1 (2 ng / mL) / Control	TGFβ-1 (10 ng / mL) / Control	ALKi / Control
ECM receptor	Angiopoietin-2	Angpt2	O35608	15	6.54±2.42	4.68±3.92	4.95±0.37	6.78±2.05	0.524	0.323	0.902
ECM receptor	Tyrosine-protein kinase receptor UFO	Axl	Q00993	5	6.72±0.39	6.48±0.43	6.07±0.32	6.71±0.26	0.518	0.090	0.977
ECM receptor	Basal cell adhesion molecule	Bcam	Q9R069	19	7.26±0.47	6.03±0.74	5.01±0.5	7.23±0.47	0.072	**0.005**	0.957
ECM receptor	CD44 antigen	Cd44	P15379	10	6.34±0.72	6.56±0.19	6.87±0.36	6.27±0.68	0.635	0.317	0.919
ECM receptor	Leukocyte surface antigen CD47	Cd47	Q61735	16	6.71±0.64	6.39±0.59	6.18±0.66	6.92±0.82	0.563	0.378	0.741
ECM receptor	CD9 antigen	Cd9	P40240	22	6.73±0.39	6.13±0.66	6.36±0.65	6.73±0.69	0.245	0.446	0.995
ECM receptor	Epidermal growth factor receptor	Egfr	Q01279	14	7.12±0.31	6.34±0.3	6.19±0.53	6.29±0.5	**0.036**	0.058	0.069
ECM receptor	Glypican-1	Gpc1	Q9QZF2	27	5.95±0.66	6.67±0.24	7.11±0.22	6.29±0.73	0.153	**0.045**	0.590
ECM receptor	Transmembrane glycoprotein NMB	Gpnmb	Q99P91	10	6.66±1.23	3.13±2.26	0.69±0.93	8.24±0.47	0.076	**0.003**	0.107
ECM receptor	Integrin alpha-2	Itga2	Q62469	26	5.11±0.41	6.7±1.02	7.39±0.96	4.7±0.47	0.067	**0.020**	0.316
ECM receptor	Integrin alpha-3	Itga3	Q62470	30	6.73±0.56	5.79±1.25	6.03±1.24	7.03±0.18	0.298	0.421	0.440
ECM receptor	Integrin alpha-5	Itga5	P11688	31	6.76±0.43	6.47±0.29	7±0.31	6.55±0.42	0.399	0.474	0.577
ECM receptor	Integrin alpha-6	Itga6	Q61739	6	3.77±3.14	4.2±3.58	6.47±0.74	3.55±2.95	0.881	0.220	0.935
ECM receptor	Integrin alpha-V	Itgav	P43406	50	6.63±0.07	6.8±0.26	6.97±0.14	6.13±0.03	0.313	0.018	**0.000**
ECM receptor	Integrin beta-1	Itgb1	P09055	51	6.58±0.14	6.6±0.57	6.93±0.38	6.51±0.02	0.954	0.205	0.484
ECM receptor	Integrin beta-3	Itgb3	O54890	17	6.33±0.31	6.59±0.76	7.37±0.51	5.64±0.56	0.610	**0.038**	0.136
ECM receptor	Integrin beta-5	Itgb5	O70309	29	6.6±0.59	6.79±0.51	7.34±0.11	5.47±0.11	0.691	0.099	**0.030**
ECM receptor	Prolow-density lipoprotein receptor-related protein 1	Lrp1	Q91ZX7	38	6.86±0.2	6.57±0.26	6.59±0.07	6.65±0.16	0.185	0.082	0.206
ECM receptor	Neurogenic locus notch homolog protein 2	Notch2	O35516	2	6.69±0.62	6.63±0.14	6.78±0.15	6.22±0.6	0.886	0.822	0.398
ECM receptor	P2X purinoceptor 4	P2rx4	Q9JJX6	17	7.19±0.33	5.71±0.29	3.75±0.9	7.69±0.15	**0.004**	**0.003**	0.072
ECM receptor	Urokinase plasminogen activator surface receptor	Plaur	P35456	17	5.41±1.4	6.68±0.11	7.5±0.6	5.03±0.86	0.191	0.076	0.710
ECM receptor	Lysosome membrane protein 2	Scarb2	O35114	47	7.21±0.06	6.05±0.69	5.83±0.82	7.21±0.03	**0.042**	0.044	0.925
ECM receptor	Syndecan-4	Sdc4	O35988	15	4.65±3.89	7.27±0.81	6.97±0.76	3.89±3.26	0.316	0.369	0.809

*N=3 biological replicates and N=2 technical replicates per condition. Significance compared to the control group was determined by unpaired Student's t-test with a p value <0.05 considered significant. SD = standard deviation. Missing values were imputed.

Supplementary Table 4. Reagents and equipment.

Description	Source / Repository	Persistent ID / URL
Cell culture reagents		
SmBM-2 Smooth Muscle Cell Growth Medium-2 BulletKit	Lonza	CC-3182
Smooth Muscle Cell Growth Medium 2 (Ready-to-use)	PromoCell	C-22062
Dulbecco's Modified Eagle Medium (DMEM)	Gibco	10938-025
Fetal bovine serum	Gibco	10500-064
L-Glutamine	Gibco	25030-024
Pen-strep	Gibco	15140-122
Trypsin-EDTA	Gibco	25300-054
Dulbecco's Phosphate Buffered Saline	Sigma-Aldrich	D8537
Cryo-SFM	PromoCell	C-29910
DMEM/F-12, no phenol red	Gibco	21041025
Engineered vascular tissue reagents		
Bovine fibrinogen	Sigma Aldrich	F8630
Bovine aprotinin	Sigma Aldrich	A1153
Bovine thrombin	Sigma Aldrich	T9549
DMEM (powdered)	Gibco	52100-021
Ultrapure Agarose	Invitrogen	16500-500

Teflon spaces	EHT Technologies GmbH	C0002
Silicone racks	EHT Technologies GmbH	C0001
Calcification reagents		
Calcium chloride dihydrate	Sigma	C7902
Sodium phosphate monobasic	Sigma	S5011
M199 Media	Sigma	M2154
In Situ Cell Death Detection Kit, Fluorescein	Merck	11684795910
DNAse II	Merck	D8764
DAPI (4',6-diamidino-2-phwnylindole, dihydrochloride)	Invitrogen	D21490
Alizarin red	Sigma-Aldrich	A5533
TGFB1 signalling reagents		
Recombinant murine TGFB1	R&D Systems	7666-MB-005/CF
SB 431542	TOCRIS	1614
Western blot materials		
Tris base	Sigma Aldrich	77-86-1
Sodium Dodecyl Sulfate	Invitrogen	15525017
Methanol (HPLC Grade)	Fischer Chemical	67-56-1

Glycerol	Sigma Aldrich	G9012
2-mercaptoethanol	Sigma Aldrich	M6250
Bromophenol blue	Sigma Aldrich	B0126
UltraPure 10X TBE Buffer	Invitrogen	15581-028
NuPAGE 4-12 % Bis Tris Gels	Thermo Fisher	NP032
MagicMark XP Western Protein Standard	Thermo Fisher	LC5800
Nitrocellulose membranes	GE healthcare	RPN2020D
TWEEN 20	Sigma Aldrich	P7949
BSA	Gibco	10938025
Sodium azide	Sigma Aldrich	S2002
ECL Western Blotting Detection Reagent	GE healthcare	RPN2209
Medical X-ray film	FUJI FILM	AUT-300-040D
Skim milk powder	SERVA	42590.02
Ponceau S solution	Sigma	P7170
Restore WesternBlot Stripping Buffer	Thermo Scientific	21059
Developer / Replenisher Concentrate	Durr NDT GmBH	CXB310A7540
Fixer / Replenisher Concentrate	Durr NDT GmBH	CXB311A7540
Chameleon® Duo Pre-Stained Protein Ladder	Li-COR®	928-60000
4X Protein Sample Loading Buffer for Western Blots	Li-COR®	928-40004

RT-qPCR reagents		
Qiazol® Lysis Reagent	Qiagen	79306
Proteinase K	Ambion	25530-015
SuperScript VILO cDNA Synthesis Kit	Thermo Fisher	11754050
Ethanol Absolute, for Molecular Biology	Fisher Chemicals	64-17-5
miRNeasy Mini Kit	Qiagen	1038703
SYBR Select Mater Mix	Applied Biosystems	01074677
Human Thoracic Aortic Smooth Muscle Cells		
Cryopreserved Primary Human Aortic Smooth Muscle Cells	PromoCell	C12532; 437Z012.2
Cryopreserved Primary Human Aortic Smooth Muscle Cells	PromoCell	C12532; 411Z027.3
Cryopreserved Primary Human Aortic Smooth Muscle Cells	Lonza	CC-2571; 369150
Deglycosylation enzymes		
a2-3,6,8,9-neuraminidase (Sialidase)	EDM Millipore	362280 (KP0012)
β1 ,4-galactosidase	EDM Millipore	362280 (KP0004)
β-N-acetylglucosaminidase	EDM Millipore	362280 (KP0013)
Chrondroitinase ABC	Sigma-Aldrich	C3667
Endo-α-N-acetylgalactosaminidase (O- glycosidase)	EDM Millipore	362280 (KP0011)
Heparinase II	Sigma-Aldrich	H6512

Keratanase	Sigma-Aldrich	G6920
PNGase F (N-Glycosidase F)	EDM Millipore	362280 (KP0001)
Mass spectrometry reagents / apparatus		
18O-Water	Tayo Nippon Sanso	FO3-0027
Urea	Fisher Scientific	11337898
Thiourea	Supelco	7979
Dithiothreitol (DTT)	Sigma Aldrich	D0632
Iodoacetamide (IAA)	Sigma Aldrich	I6125
Trypsin, MS Grade	Thermo Scientific	Trypsin, MS Grade
Triethylammonium bicarbonate (TEAB)	Sigma Aldrich	T7408
Trifluoroacetic acid (TFA)	Thermo Scientific	85183
Acetonitrile (ACN)	Thermo Scientific	51101
Formic acid (FA)	Thermo Scientific	ES803
Macro Spin Column C-18 96-well plate	Harvard Apparatus	74-5657
Nanoflow LC system	Thermo Scientific	UltiMate 3000 RSLC nano
EASY-Spray Source	Thermo Scientific	ES081
EASY-Spray PepMap® RSLC C18, 2µm 100 Å, 75µm x 50 cm column	Thermo Scientific	ES803A
C18 trap cartridge	Thermo Scientific	160454
Hybrid mass spectrometer	Thermo Scientific	Q Exactive HF

Antibodies / Stainings		
Alizarin red	Sigma-Aldrich	A5533
Anti-Versican antibody (versikine)	Abcam	ab18345
Anti-Versican antibody (full-length versican)	Sigma Aldrich	AB1033
Anti-Alpha smooth muscle actin antibody	Abcam	ab21027
Anti-Beta actin antibody	Sigma Aldrich	A1978
Anti-Periostin antibody	Santa Cruz	Sc-67233
Anti-Transgelin	Abcam	ab14106
Anti-Alpha smooth muscle actin antibody	Abcam	ab5694
Peroxidase Mouse Anti-Rabbit IgG	Jackson Immuno Research	211-032-171
Peroxidase Goat Anti-Mouse IgG	Jackson Immuno Research	115-035-174
Peroxidase Rabbit Anti-Rat IgG	Dako	P0450
Peroxidase Mouse Anti-Goat IgG	Jackson Immuno Research	205-032-176
Alex Fluor 546 Goat Anti-Rabbit IgG	Invitrogen	A-11035
Reagents for Immunohistochemistry		
OCT Embedding Matrix for Frozen Sections	Cell Path	KMA-0100-00A
Sucrose for molecular biology	Sigma	S0389
Dako Pen	Dako	S2002
VectaMount® AQ Aqueous Mounting Medium	Vector Laboratories	H-5501

10% neutral buffered formalin	CellPath	3811775
Tissue-Tek® Cryomold®	Sakura Finetek	4565
SuperfrostPlus Adhesion Microscope Slides	Epredia	J1820AMNZ
Donkey serum	Sigma	D9663
2-methyl butane	Sigma-Aldrich	277258
Dented microscope slides	Carl Roth	H884.1
Glass coverslips	Hirschmann	8000112
Paraformaldehyde	Sigma-Aldrich	158127
General laboratory reagents		
Millex-GP Filter, 0.22 µm, PES 33 mm, non-sterile	Merck Millipore	SLGP033NS
Ultrapure Milli-Q® water	Merck	C85358
10X PBS	Lonza	51226
Micro BCA protein Assay Kit	Thermo Scientific	23235
Lysing Matrix D	MP Biologicals	6913050
Hanks’ Balanced Salt solution (HBSS)*	Sigma Aldrich	H6648
Acetone	Sigma Aldrich	90872
cOmplete Mini	Roche	1183617001
PhosStop	Roche	4906837001
10x Cell lysis buffer	Cell Signaling Technologies	9803

Tris-hydrochloride	Sigma-Aldrich	T3253
Sodium chloride	Sigma-Aldrich	S9888
EGTA	Sigma-Aldrich	E5134
EDTA	Sigma-Aldrich	E3889
Triton-X 100	Sigma-Aldrich	X100
Sodium dodecyl sulfate	Genomic Solution	80-0175
Neubauer chamber	Hawksley	BS.748
Amicon Ultra-0.5 Centrifugal Filter Unit, 3 kDa cut-off columns	Millipore	UFC500396
General Laboratory Equipment		
Bravo automated liquid handling platform	Agilent Technologies	04730-201
Veriti 96-well Thermal Cycler	Applied Biosystems	9902
Microplate reader	Tecan	Infinite M200 Pro
Nanodrop® spectrophotometer	Thermo Fisher Scientific	ND1000
ViiA 7 Real-Time PCR system	Applied Biosystems	4453552
OPTIMAX 2021 X-ray film processor	PROTEC GmBH	1160-1-0000
SpeedVac Concentrator	Thermo Fisher Scientific	SPD131DDA
Vacuum Pump	Thermo Fisher Scientific	OFP400
Refrigerated Vapor Trap	Thermo Fisher Scientific	RVT5105
Infrared Imaging System	LI-COR	Odyssey CLx

50 mL centrifuge tube	Greiner	227261
NuncEasYFlask Cell Culture Flasks	Thermo Fisher Scientific	156367, 156499, 159920
CoolCellLX Cell Freezing Container	PromoCell	C-29910
CRYO.S. 2 mL Cryovial	Greiner	122263
Bench Top Autoclave	Astell	AMB230N
Cryostat	CryoStar	NX70
Tissue homogeniser	Bertin Instruments	Precellys 24
6 Well Cell Culture Plate	Greiner	657 160
12 Well Cell Culture Plate	Greiner	665 180
48 Well Cell Culture Plate	Greiner	677 180
Cell culture incubator	Eppendorf	Galaxy 170s
1.5 mL Micro tube	Sarstedt AG & Co.,	72.706.700
Graduated Skirted Tubes	STARLAB	17166
Amicon Ultra-0.5 Centrifugal Filter Unit	Millipore	UFC500396
Eppendorf Centrifuge	Eppendorf	5920 R
Biological safety cabinet	Kendro	Herasafe™ KS 12
Universal testing instrument	Instron	5569A
Vortex Mixer	Avantor	VWR, VV3
Microscopes		

Digital Color Camera	Leica	DFC310
Inverted Microscope	Nikon	Eclipse Ti-E
Software		
ImageJ	Open Source	1.52
NIS-Elements	Nikon	5.21
R Studio	Open Source	1.2.1335
Microsoft Office	Microsoft	16.56
Prism	GraphPad	9.31
Proteome Discover	Thermo Scientific	2.2.0.0388
Bluehill software	Instron	4.13
Cytoscape	Open Source	3.9.1
Cytoscape stringApp plugin	Open Source	1.7.0

Supplementary Table 5. SYBR green primers for qPCR analysis.

Target	Species	Target template	Forward primers (5'-3')	Reverse primer (3'-5')	Product length
Api5	Mus musculus	NM_001305258.2	ATTGTCCTGGAAACCTGTGC	AAGGCTCCTCTCTGCTCATAAT	189
Vcan	Mus musculus	NM_001081249.1	TGCAAGAAGGGAACAGTTGCT	CCTAGGCACCGGATAGTTGG	158
Adamts5	Mus musculus	NM_011782.2	CCTGTTCACCCGAGAGGATT	ATATGGTCCCAACGTCTGCC	74
Adamts1	Mus musculus	NM_009621.5	TTGAATGGTGTGAGTGGCGA	CATCAAACATTCCCCGTGTCC	135
Tagln	Mus musculus	NM_011526.5	GCCTTTAAACCCCTCACCCAG	TCACCAATTTGCTCAGAATCACAC	307
Smthn	Mus musculus	NM_001159284.1	GAGTCCAGGGTACGTTGCTG	CCCAGGGTATTTTGCTCTCAGT	153
Mgp	Mus musculus	NM_008597.4	GCAACCCTGTGCTACGAATCT	TAGTCATCGCAGGCCTCTCT	182
Bglap	Mus musculus	NM_007541.3	GAACAGACAAGTCCCACACAGC	TCAGCAGAGTGAGCAGAAAGAT	79

Supplementary Fig 1.

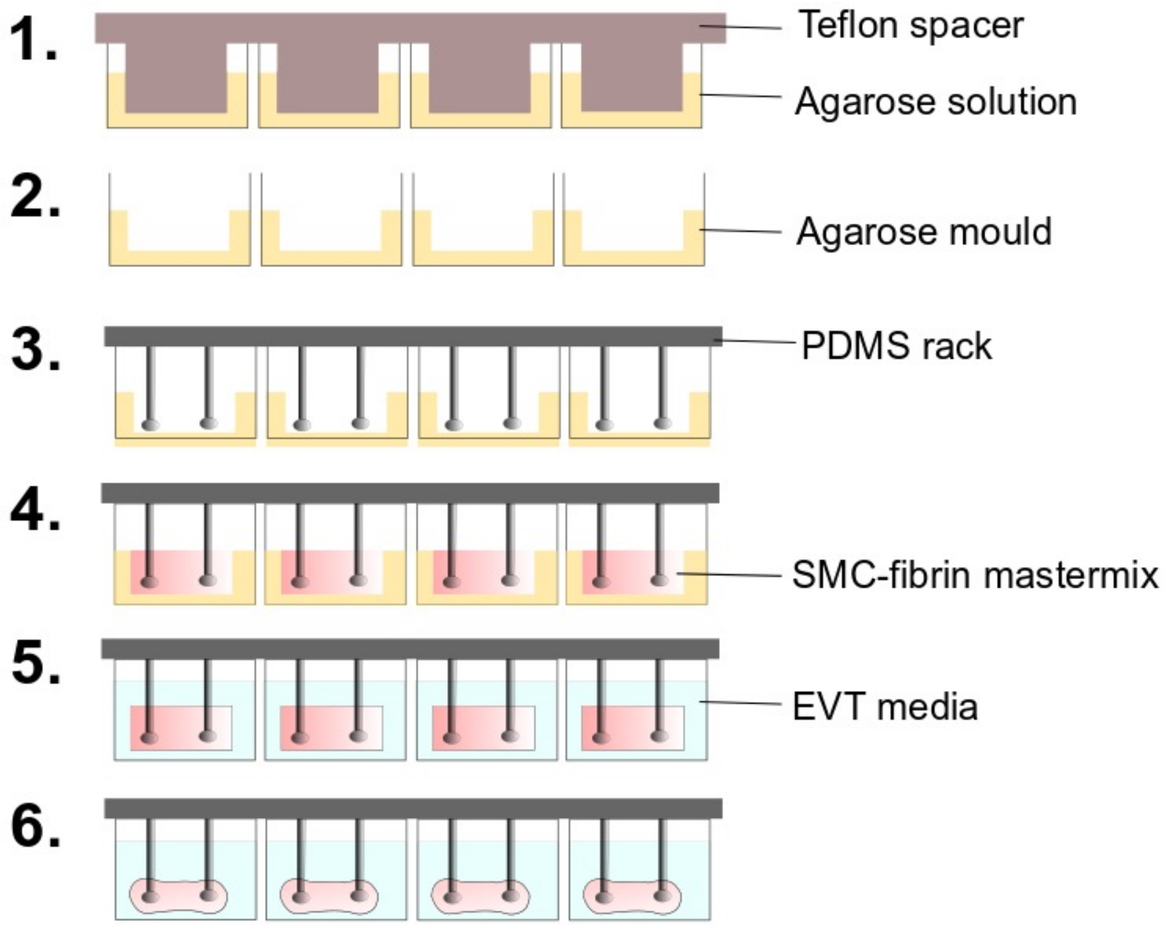

Supplementary Fig.1. Formation of EVTs. The schematic shows stepwise formation of EVTs in four wells of a 24-well plate format. 1. Pre-warmed liquid agarose solution was poured into wells and a Teflon spacer was placed on top. 2. After 10 minutes at room temperature, the Teflon spacer was removed to reveal the set agarose moulds. 3. A polydimethylsiloxane (PDMS) rack was placed into the agarose moulds. 4. The SMC-fibrinogen master mix was pipetted into the agarose moulds in between the PDMS rack. On addition of thrombin fibrin polymers form. EVTs are then incubated for 1 hour at 37 °C and 5% CO_2 to allow for fibrin polymerisation and the EVTs to "set". 5. Set EVTs are suspended on the PDMS rack and can be removed from the agarose mould and placed into a new 24-well plate containing fresh EVT.

Supplementary Fig 2.

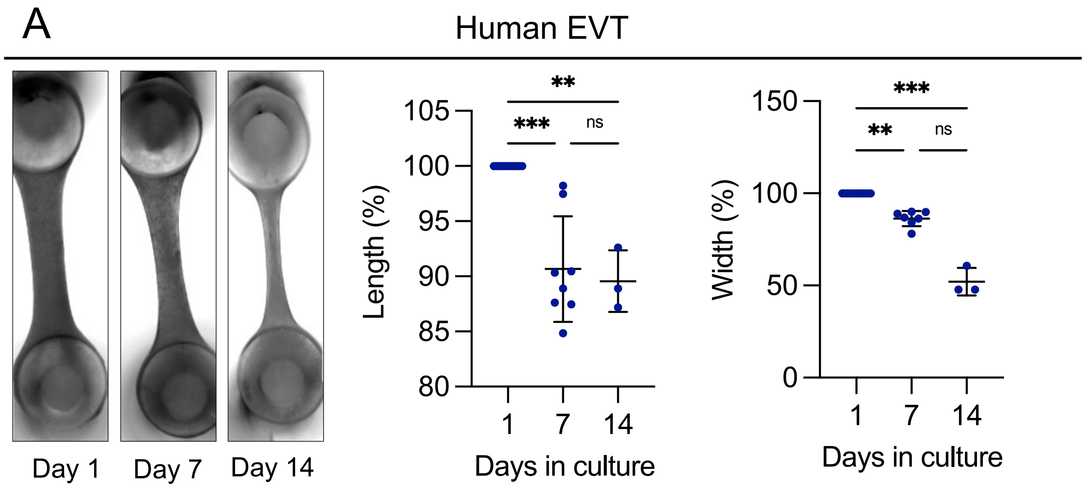

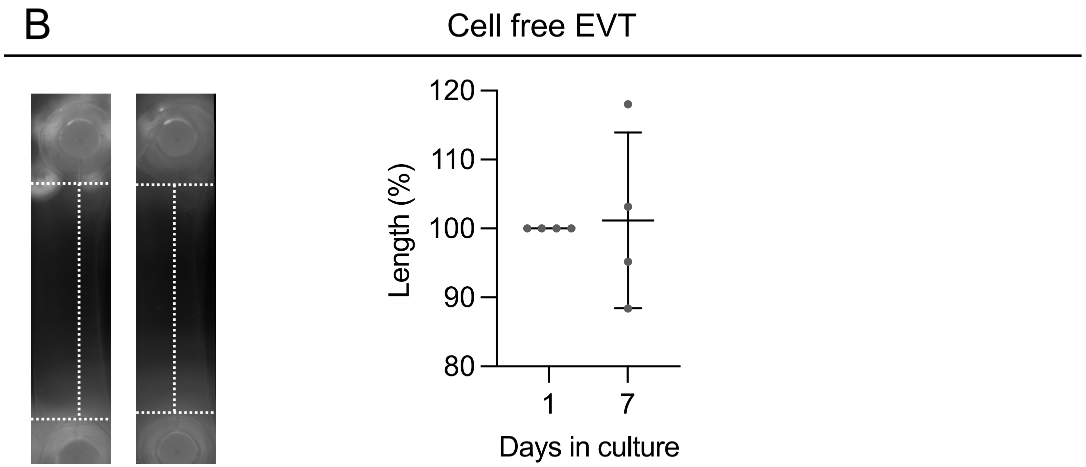

Supplementary Fig.2. Length and width remodelling of murine and human EVTs. Over prolonged culture EVTs remodel to become shorter and thinner over time. (**A**) Representative inverted light microscope images and quantification of EVT length and width of human SMC EVTs at day 1, 7 and 14 of culture. N≥3 technical replicates. Significance was determined by one-way ANOVA with Dunn's correction for multiple comparisons, a p value <0.05 was considered significant. (**B**) Representative inverted light microscope images and length quantification of cell-free EVTs at day 1 and day 7. N=4 technical replicates. Significance was determined by a paired Student's T-test. A p value <0.05 was considered significant.

Supplementary Fig 3.

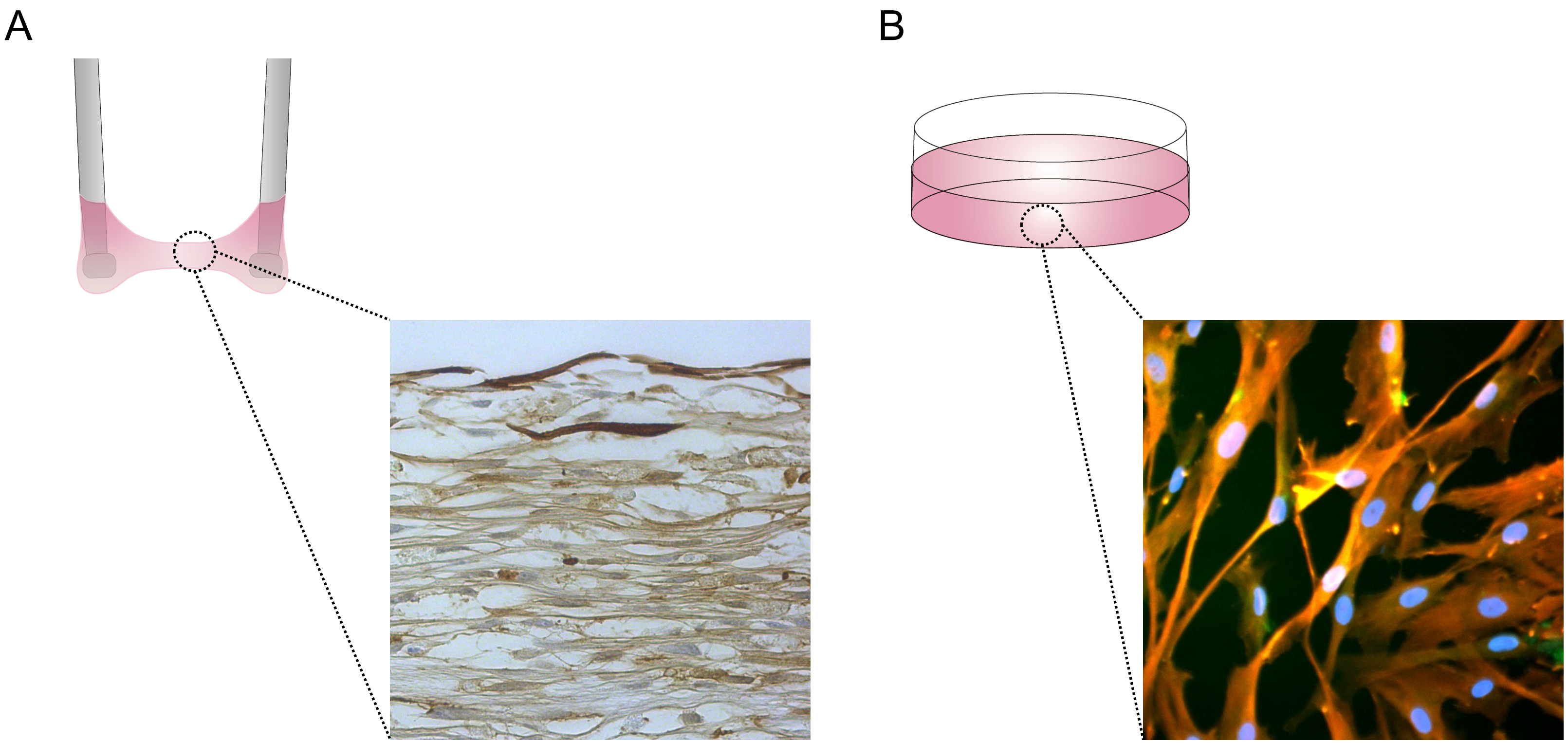

Supplementary Fig.3. Tissue-like architecture within EVTs. (**A**) Human primary SMCs (male, aged 43) cultured within EVTs for 7 days. Longitudinal paraffin sections were stained with an antibody raised against TAGLN. (**B**) SMCs from the same donor were seeded onto a glass slide and cultured for 24 hours before fixation and immunofluorescence imaging. The 2D cultured SMCs were stained with antibodies raised against TAGLN (red) and ACTA2 (green). Nuclei were stained with DAPI (blue).

Supplementary Fig 4.

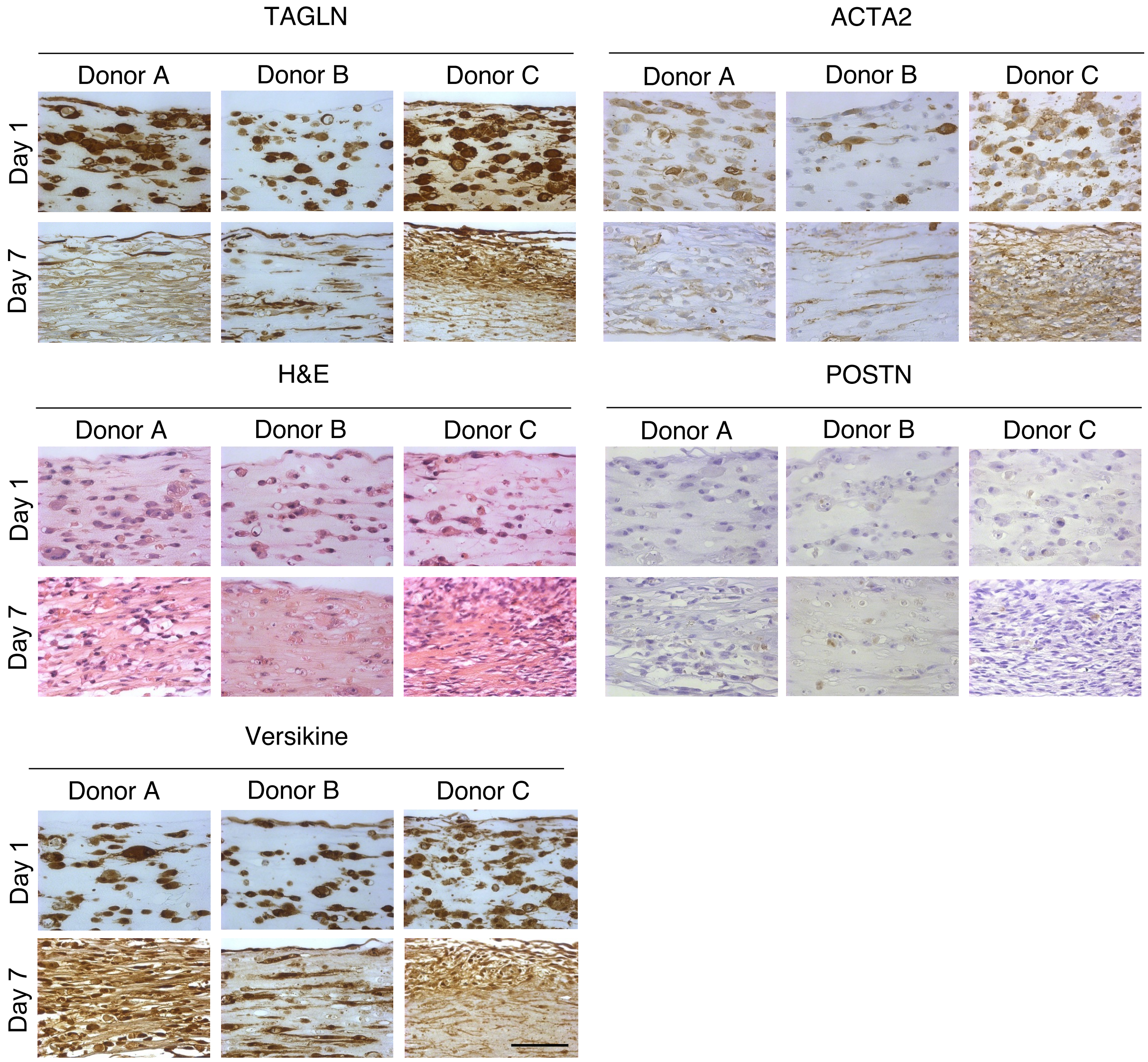

Supplementary Fig.4. Immunohistochemical analysis of human EVTs. Human EVT longitudinal sections from three different donors were stained for transgelin (TAGLN), smooth muscle actin (ACTA2), hematoxylin and eosin (H&E) and periostin (POSTN) and versikine after 1 and 7 days of culture. Scale bar = 100 µm.

Supplementary Fig 5.

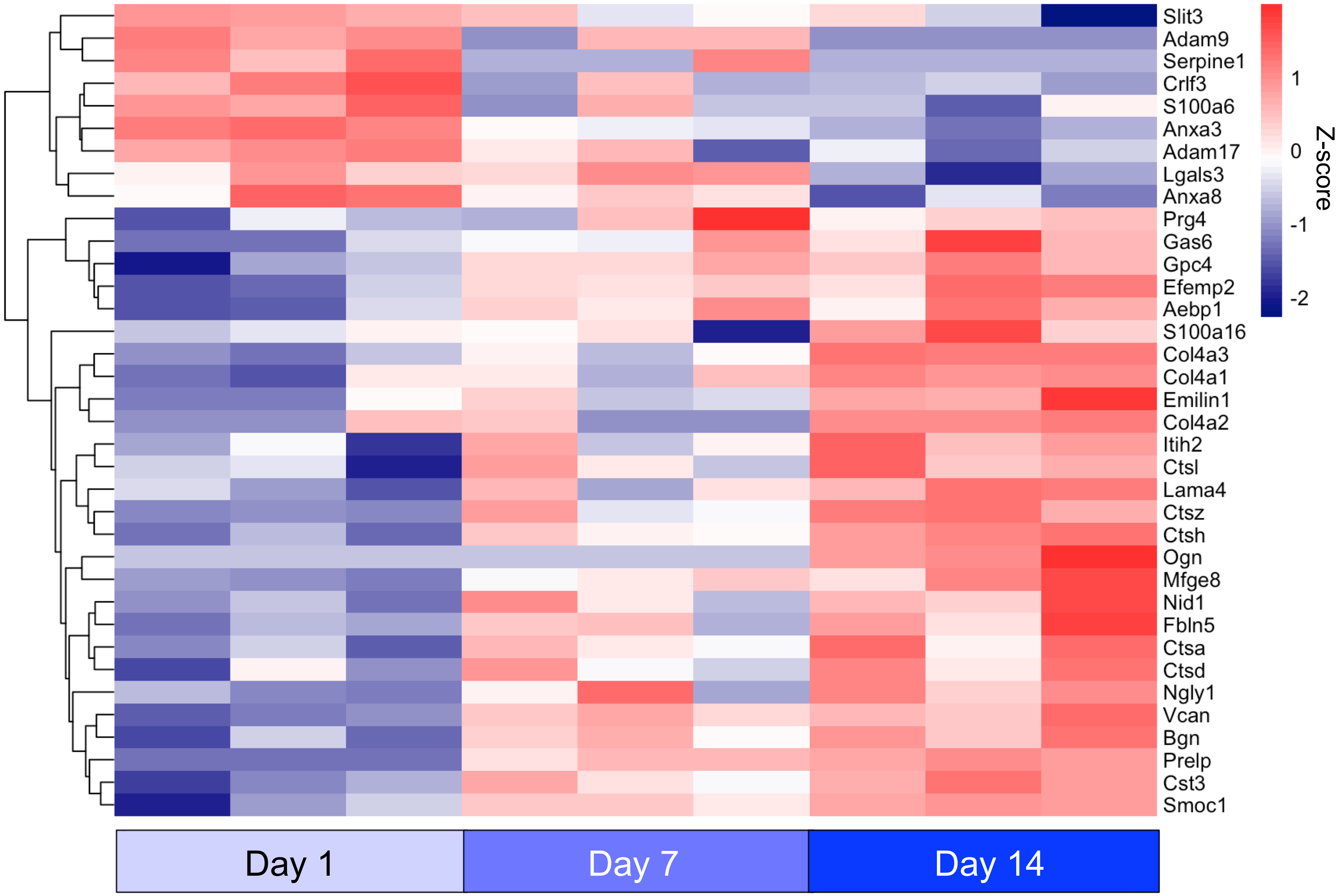

Supplementary Fig.5. Accumulation of ECM proteins within EVTs. Heatmap of significantly changing ECM proteins at day 1, 7 or 14 of EVT culture using z-scores from log2 transformed abundances. Clustered heatmaps were generated from protein abundances using the pheatmap R package version 1.0.12. N=3 biological replicates, N=2 technical replicates.

Supplementary Fig 6.

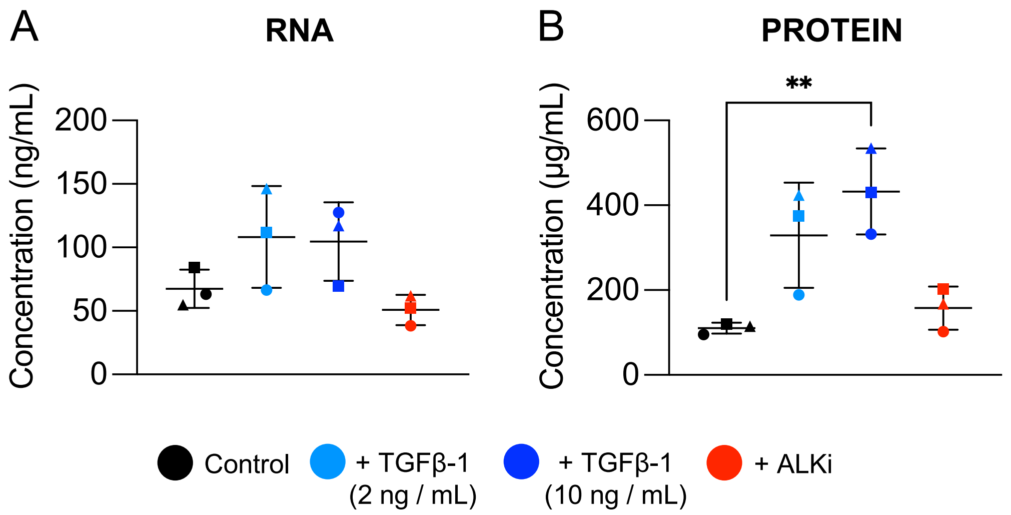

Supplementary Fig.6. (**A**) Total concentration of RNA extracted from EVTs, and (**B**) total concentration of protein extracted from EVTs after seven days of culture under control conditions or treatment with TGFβ-1 or ALKi as indicated. N=3 biological replicates presented as an average of N=2 technical replicates. Error bars show SD. Significance compared to the control group was determined by ordinary one-way ANOVA with Dunnett's multiple comparison test. A p value of <0.05 was considered significant.

Supplementary Fig 7.

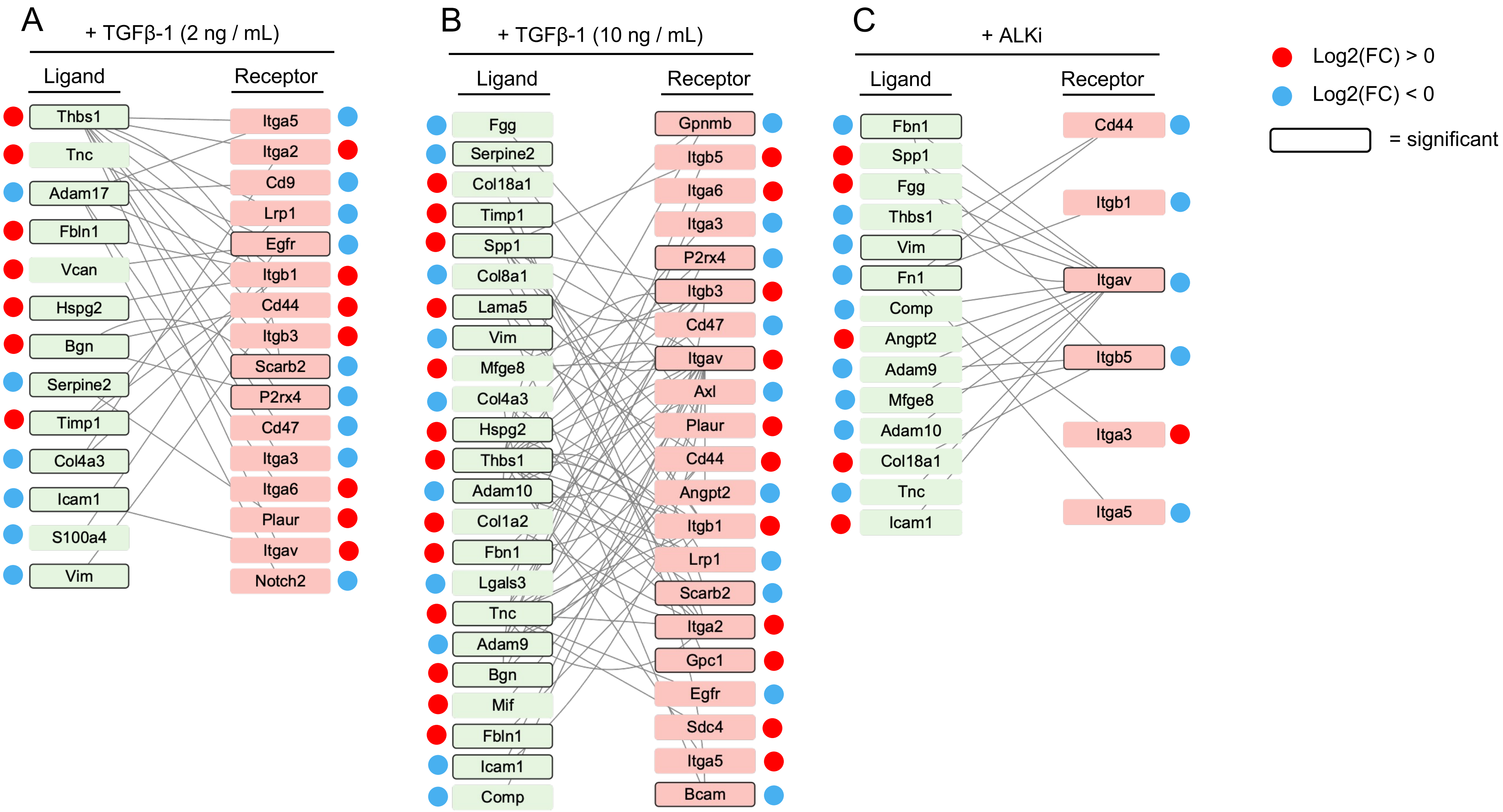

Supplementary Fig.7. Detection of ECM ligand and receptor pairs within EVTs. Receptors and ligands detected in the LC-MS/MS analysis of treated EVT lysates were selected based on a known or putative interaction using the open access CellTalkDB murine database[41]. The figure presents ligand-receptor pairs where either a ligand or receptor in the pair is significantly altered after treatment with (**A**) TGFβ-1 (2 ng / mL), (**B**) TGFβ-1 (10 ng / mL), (**C**) ALKi (10 µm) relative to the control untreated group. Lines between ligands (green) and receptors (pink) demonstrate interaction partners. Circles reflect the directionality of the fold change in protein abundance relative to the untreated control EVTs: red circles indicate an upregulation whereas blue circles represent a downregulation. Each circle represents the average protein abundance for N=3 biological replicates and N=2 technical replicates. A black border indicates a significant change in protein abundance relative to the control group. Significance relative to the control group was determined by an unpaired Student's T-test with a p value <0.05 considered significant. FC = fold change. Figure created using Cytoscape.
